# Vitamin D Receptor Genotype, Tumor Expression, and Outcomes After Vitamin D Supplementation in Digestive Tract Cancer: An Exploratory Post Hoc Analysis of the AMATERASU Trial

**DOI:** 10.3390/cancers18213570

**Published:** 2026-11-06

**Authors:** Tadashi Matsushima, Akitaka Sasaki, Taisuke Akutsu, Hironori Ohdaira, Yutaka Suzuki, Mitsuyoshi Urashima

**Affiliations:** 1 Division of Molecular Epidemiology, The Jikei University School of Medicine, 3-25-8, Nishi-Shimbashi, Minato-Ku, Tokyo 105-8461, Japan; tadashi.m@jikei.ac.jp (T.M.); h25ms-sasaki@jikei.ac.jp (A.S.); t-akutsu@jikei.ac.jp (T.A.); 2 Department of Surgery, The Jikei University School of Medicine, 3-25-8, Nishi-Shimbashi, Minato-Ku, Tokyo 105-8461, Japan; 3 Department of Surgery, International University of Health and Welfare Hospital, 537-3 Iguchi, Nasushiobara 329-2763, Tochigi, Japan; ohdaira@ihwg.jp (H.O.); yutaka@ihwg.jp (Y.S.)

**Keywords:** vitamin D supplementation, vitamin D receptor, FokI polymorphism, tumor VDR expression, digestive tract cancer, relapse-free survival, predictive biomarkers, treatment effect modification, randomized controlled trial

## Abstract

**Background/Objectives:** Vitamin D supplementation effects may vary according to receptor genotype and tumor receptor abundance. We examined whether treatment effects differed according to FokI genotype and tumor nuclear vitamin D receptor (VDR) expression after surgery for digestive tract cancer. **Methods**: This exploratory post hoc analysis used data from the double-blind AMATERASU randomized trial (UMIN000001977), which assigned 417 patients with resected stage I–III digestive tract cancers to vitamin D_3_ (2000 IU/day) or placebo. FokI genotype was determined by sequencing and nuclear VDR expression by immunohistochemistry. Unadjusted Cox analyses were supplemented by pooled treatment-by-subgroup interaction models adjusted for age, sex, and pathological stage, with baseline hazards stratified by tumor site. **Results:** The joint analysis included 359 patients (213 vitamin D; 146 placebo). VDR expression varied across tumor sites. Among patients with the FokI CC genotype and VDR expression in quintiles 2–5 (*n* = 110), the unadjusted treatment hazard ratio (HR) for relapse or death was 0.30 (95% confidence interval [CI], 0.12–0.73), compared with 1.02 (95% CI, 0.61–1.71) in the complementary subgroup (*n* = 249; nominal interaction *p* = 0.02). After adjustment, the HR in the CC/VDR Q2–5 subgroup was 0.44 (95% CI, 0.18–1.10; interaction *p* = 0.211). For all-cause mortality, the adjusted HR was 0.46 (95% CI, 0.14–1.51; interaction *p* = 0.135). **Conclusions:** The unadjusted exploratory findings were attenuated after accounting for clinicopathological factors. Differential supplementation benefit according to the joint classification was not established. Independent validation is required before clinical use.

## Simple Summary

Vitamin D supplementation may not benefit all patients with digestive tract cancer equally. We investigated whether genetic differences in the vitamin D receptor and the amount of this receptor in tumor cells could help identify patients more likely to benefit. We analyzed genetic and tumor samples from 359 patients who had undergone surgery and participated in a randomized trial comparing vitamin D with placebo. An apparent benefit was observed in unadjusted analyses among patients with a particular receptor genotype and tumor receptor levels above the lowest fifth. However, after accounting for age, sex, cancer stage, and tumor site, the treatment effect and its difference from that in the remaining patients were no longer statistically clear. These exploratory findings do not establish that this classification predicts supplementation benefit. Further studies are needed before it can guide treatment decisions.

## 1. Introduction

Cancer remains a substantial public health burden worldwide, with an estimated 20.6 million new diagnoses and 9.8 million deaths in 2024 [1]. Demographic projections from the International Agency for Research on Cancer, the cancer agency of the World Health Organization, indicate that these annual numbers could reach approximately 34.4 million and 17.5 million, respectively, by 2050 if age-specific incidence and mortality rates remain unchanged [1,2]. In Japan, based on 2023 incidence data, the lifetime probability of being diagnosed with cancer is 61.1% for men and 50.1% for women; based on 2024 mortality data, the corresponding probability of dying from cancer is 24.4% and 17.2%, respectively [3]. Although Japan’s annual number of new cancer cases is projected to change little between 2024 and 2050, annual cancer deaths are projected to increase from approximately 455,000 to 516,000 [2]. These substantial and persistent burdens highlight the need for effective cancer prevention and additional strategies to improve outcomes after diagnosis.

Vitamin D has attracted interest because of its effects on cancer cells and their surrounding environment. In experimental colon cancer models, its active metabolite, 1,25-dihydroxyvitamin D_3_, promotes epithelial differentiation through vitamin D receptor (VDR)-dependent induction of E-cadherin and inhibition of β-catenin signaling [4]. Active vitamin D also reduced the ability of fibroblasts isolated from colorectal tumors to promote cancer cell migration, and higher stromal VDR expression was associated with favorable clinical outcomes [5]. Potential effects extend to host immunity: vitamin D enhanced antitumor immunity and responses to immune checkpoint blockade through microbiome-dependent mechanisms in mice [6], and a small human mechanistic study reported increased rituximab-mediated cellular cytotoxicity after correction of vitamin D deficiency [7]. An ancillary study of the VITamin D and OmegA-3 TriaL (VITAL) also found reduced leukocyte telomere attrition with vitamin D_3_ supplementation, although the implications for cancer outcomes remain uncertain [8]. These findings provide biological grounds for investigating vitamin D supplementation in cancer.

Clinical findings have, however, been inconsistent. In VITAL, vitamin D_3_ supplementation at 2000 IU/day did not significantly reduce invasive cancer incidence [9], although a secondary analysis reported a lower incidence of metastatic or fatal cancer [10]. Meta-analyses have suggested a possible reduction in cancer mortality, with no corresponding reduction in overall cancer incidence [11,12]. In the postoperative setting, the AMATERASU randomized trial evaluated vitamin D_3_ supplementation at 2000 IU/day in 417 patients with resected stage I–III digestive tract cancers. It did not demonstrate a statistically significant improvement in relapse-free survival in the overall population (hazard ratio [HR] for relapse or death, 0.76; 95% confidence interval [CI], 0.50–1.14) [13]. These results leave the clinical benefit of supplementation uncertain and raise the question of whether treatment effects vary according to host and tumor characteristics.

One potential source of variation is the capacity to respond to vitamin D through VDR. This receptor regulates gene transcription in partnership with the retinoid X receptor, linking vitamin D and retinoid signaling. In a previous exploratory post hoc analysis of AMATERASU, we observed a lower hazard of relapse or death with supplementation among patients whose serum vitamin A concentrations were within deciles 6–8 [14]. Although this finding requires independent validation, it motivated further investigation of factors that may modify vitamin D responsiveness.

Genetic variation in VDR is one such factor. The FokI start-codon polymorphism (rs2228570) produces receptor isoforms containing either 424 or 427 amino acids. The shorter, C-allele-associated isoform has shown greater transcriptional activity and more efficient interaction with transcription factor IIB in experimental studies [15,16]. It also conferred greater sensitivity to the growth-inhibitory effects of active vitamin D in engineered breast cancer cells [17]. Functional differences have been reported in immune-cell signaling [18], but their magnitude and direction depend on the experimental system and pathway examined [18,19]. An observational study of advanced non-small-cell lung cancer associated the FokI CC genotype with longer overall survival [20]. However, neither experimental functional differences nor prognostic associations establish whether FokI predicts benefit from vitamin D supplementation.

Receptor abundance may also influence responsiveness. VDR expression has been documented in gastric and colorectal cancers, with differences according to cellular compartment and tumor characteristics [5,21]. Experimental differentiation responses in VDR-expressing, but not VDR-deficient, colon cancer cells suggest that low receptor abundance may limit direct responses to active vitamin D [4]. These observations support evaluating genotype-inferred functional potential and tumor receptor abundance jointly, while recognizing that stromal and immune mechanisms may also contribute.

In this exploratory post hoc analysis of the AMATERASU randomized trial, we examined whether the effect of vitamin D_3_ supplementation on relapse or death differed between patients with both the FokI CC genotype and tumor nuclear VDR expression in quintiles 2–5 and those in the complementary subgroup. We evaluated unadjusted associations and additional analyses accounting for age, sex, pathological stage, and primary tumor site; the findings do not establish a clinically usable predictive classification.

## 2. Materials and Methods

### 2.1. AMATERASU Trial and Study Population

This exploratory post hoc biomarker analysis used data and specimens from the AMATERASU randomized, double-blind, placebo-controlled trial conducted at International University of Health and Welfare Hospital, Japan (UMIN000001977). The parent trial enrolled patients aged 30–90 years with resected stage I–III epithelial cancers of the digestive tract and randomly assigned them in a 3:2 ratio to oral vitamin D_3_ (2000 IU/day) or matching placebo after surgery. Detailed eligibility criteria, randomization procedures, and follow-up methods have been reported previously [13]. The present analysis retained the original randomized treatment assignments.

The primary endpoint was relapse-free survival, defined as the time from randomization to the first documented cancer recurrence or death from any cause. Overall survival, defined as the time from randomization to death from any cause, was the secondary endpoint.

The parent trial was approved by the institutional review boards of International University of Health and Welfare Hospital (13-B-263; 4 December 2009) and The Jikei University School of Medicine (21–216 [6094]; 1 January 2010). All participants provided written informed consent.

### 2.2. FokI Genotyping

This analysis used archived DNA samples and FokI genotype data that had been collected and generated during the conduct of the AMATERASU trial [13]; no additional blood collection, DNA extraction, or genotyping was performed for the present analysis. Peripheral blood samples were collected at International University of Health and Welfare Hospital and sent to SRL Inc. (Hachioji, Tokyo, Japan) for genomic DNA extraction. Purified genomic DNA was transferred to the Division of Molecular Epidemiology, The Jikei University School of Medicine, and stored at −80 °C.

A DNA fragment encompassing the VDR FokI start-codon polymorphism (rs2228570; historically designated rs10735810) was amplified by polymerase chain reaction using the forward primer 5′-CTCCGAAGGCACTGTGCTCAGGCCT-3′ and reverse primer 5′-ATGGAAACACCTTGCTTCTTCTCCCTC-3′. PCR conditions consisted of initial denaturation at 98 °C for 1 min, followed by 30 cycles of denaturation at 98 °C for 10 s and combined annealing and extension at 68 °C for 4 min; products were then held at 16 °C. Genotypes were determined by direct sequencing of the amplified products.

Genotypes were recorded as CC, CT, or TT. For the joint biomarker analysis, patients with the CC genotype were compared with those with the CT or TT genotype, based on experimental evidence concerning functional differences between the shorter, C-allele-associated VDR isoform and the longer, T-allele-associated isoform [15,16,17,18,19].

Further details of the original genotyping quality-control procedures and the blinding status of personnel performing genotype determination could not be confirmed from the available information. Accordingly, an assay-specific technical success rate and repeat-assay concordance could not be established. Hardy–Weinberg equilibrium was assessed among all 402 participants with available genotypes using a Pearson goodness-of-fit chi-square test with one degree of freedom; expected counts were calculated from allele frequencies estimated in these participants.

### 2.3. Tumor VDR Immunohistochemistry

This analysis reused tumor nuclear VDR immunohistochemistry data that had been generated from surgical specimens obtained during the AMATERASU trial and reported previously in a post hoc analysis of the trial [21]. No additional tissue sampling, tissue microarray construction, immunohistochemical staining, or pathological scoring was performed for the present analysis.

As described previously [21], representative formalin-fixed, paraffin-embedded tumor areas were selected after reviewing hematoxylin-eosin-stained sections, with preference given to viable invasive tumor and avoidance of necrotic regions. Tissue microarrays were constructed using tumor cores measuring 5 mm in diameter. After heat-induced antigen retrieval in pH 9.0 buffer and blockade of endogenous peroxidase activity, sections were incubated with a rabbit monoclonal anti-VDR antibody (clone D2K6W; Cell Signaling Technology; dilution, 1:200). Immunoreactivity was visualized using a polymer-based horseradish peroxidase detection system with diaminobenzidine and hematoxylin counterstaining.

Nuclear staining was evaluated in tumor epithelial or glandular components, as applicable. Staining intensity was scored as 1 (no immunostaining), 2 (weak), 3 (moderate), or 4 (strong). For mixed intensities, the higher intensity was used according to the previously reported ≥20% criterion [21]. The percentage of immunoreactive tumor-cell nuclei (0–100%) was multiplied by intensity to generate a semiquantitative score from 0 to 400. Two pathologists independently assessed the slides while blinded to patient information; their mean score was used. A Spearman correlation coefficient of 0.83 between their scores was reported previously [21]. Cases without adequate tissue or interpretable staining were considered unavailable.

No positive or negative staining controls were included in the VDR immunohistochemistry procedure. Formal measures of absolute interobserver agreement, such as an intraclass correlation coefficient, and agreement of the biomarker categories using kappa statistics were not available.

### 2.4. Definition of the Joint Biomarker Cohort

FokI genotype and tumor VDR expression were evaluated as complementary biomarker dimensions. Patients with results available for both assessments constituted the joint biomarker cohort. Tumor VDR scores were divided into quintiles, from Q1 (lowest expression) to Q5 (highest expression), according to their distribution among the 372 patients with available VDR measurements. The primary joint analysis compared patients with the FokI CC genotype and tumor VDR expression in Q2–Q5 with the complementary group, defined as patients with a CT/TT genotype, tumor VDR expression in Q1, or both. Treatment effects were estimated according to the original randomized assignment, and the treatment-by-subgroup interaction was used to assess whether the association between vitamin D supplementation and relapse-free survival differed between the two groups.

The observed VDR score ranges in these 372 patients were 15–85 for Q1 (*n* = 75), 90–150 for Q2 (*n* = 74), 151–212 for Q3 (*n* = 74), 212.5–290 for Q4 (*n* = 78), and 295–400 for Q5 (*n* = 71). The existing assignments corresponded to scores ≤ 85, >85 to ≤150, >150 to <212.5, ≥212.5 to ≤290, and >290, respectively. Thus, the observed score of 212.5 belonged to Q4. These observed ranges and classification boundaries are distinguished because gaps between observed scores do not define additional categories. The existing assignments were applied unchanged to the 359-patient joint biomarker cohort.

### 2.5. Statistics

Baseline characteristics were summarized by joint biomarker subgroup and randomized treatment assignment. Continuous variables were presented as means and standard deviations, and categorical variables as counts and percentages. Continuous-variable summaries used available observations, with variable-specific denominators reported for missing values. The distribution of VDR expression quintiles across anatomical tumor sites was described using counts and within-site percentages among the 372 patients with available VDR measurements.

Baseline age, sex, pathological stage, primary tumor site, and randomized treatment assignment were also compared descriptively between patients included in the joint biomarker analysis and those excluded because of missing biomarker data (Table S1).

The association between FokI genotype and relapse or death was examined among the 402 patients with genotype data using an unadjusted Cox proportional hazards model, with TT as the reference category.

The main joint biomarker analyses included 359 patients with both biomarkers available. Nelson–Aalen cumulative hazard curves were used to describe relapse or death and all-cause mortality by treatment assignment within each joint biomarker subgroup. Unadjusted treatment HRs and 95% CIs were estimated separately within each subgroup using Cox models with treatment assignment as the sole covariate. For each endpoint, treatment-effect heterogeneity was assessed in an unadjusted pooled Cox model containing treatment assignment, subgroup membership, and their interaction. The interaction was evaluated using a one-degree-of-freedom Wald test.

Additional multivariable analyses for relapse-free survival and overall survival used pooled Cox models containing treatment assignment, joint biomarker subgroup, their interaction, age as a continuous variable, and sex and pathological stage (I, II, or III) as categorical variables. Baseline hazards were stratified by primary tumor site (esophageal, gastric, small intestinal, or colorectal), with common covariate coefficients across biomarker subgroups. Site stratification avoided estimating a separate site coefficient for the two patients with small-intestinal cancer, neither of whom experienced an event. This event-free stratum contributed no information to estimation of the regression coefficients through the partial likelihood. Subgroup-specific adjusted treatment HRs and 95% CIs were obtained from linear combinations of the treatment and interaction coefficients, and interaction *p*-values from one-degree-of-freedom Wald tests. The Breslow method was used for tied event times.

The proportional hazards assumption in the adjusted models was assessed using Schoenfeld residual tests, both globally and for individual model terms. A sensitivity analysis for relapse-free survival stratified baseline hazards jointly by tumor site and pathological stage, while adjusting for age and sex and retaining treatment, biomarker subgroup, and their interaction. This allowed stage-specific baseline hazards without assuming proportional hazards for stage. The additional adjusted analyses and this sensitivity analysis were post hoc.

Additional post hoc models treated the nuclear VDR score continuously, scaled per 100 points, with treatment, CC versus CT/TT genotype, all pairwise interactions, and their three-way interaction. Both endpoints used the 359 complete cases. Models adjusted for age, sex, and stage and stratified baseline hazards by tumor site. Schoenfeld residual tests assessed proportional hazards globally and by model term. An RFS sensitivity model instead stratified jointly by tumor site and stage, retaining age and sex. Within-genotype ratios of treatment HRs per 100-point increase in VDR were estimated by linear combinations; a two-degree-of-freedom Wald test jointly assessed the treatment-by-VDR and three-way terms.

In the continuous OS model, a targeted sensitivity analysis added the product of treatment, CC genotype, VDR score per 100 points, and ln(time/3 years), allowing the three-way coefficient to vary with log time. Fit was compared with the constant-coefficient model using a one-degree-of-freedom partial-likelihood ratio test. Influence on the three-way coefficient was assessed using approximate case-deletion DFBETAs divided by its full-model standard error. For each endpoint, the model was refit after omitting the single case with the largest absolute standardized influence; RFS used the site-and-stage-stratified model and OS the site-stratified model. These targeted diagnostics do not assess influence on every model parameter, and all patients remained in the primary analyses.

Nonlinearity was explored with a restricted cubic spline using three knots at VDR scores 55, 180, and 322.5, selected by the Stata default percentile rule within the 359 complete cases. The two spline basis variables replaced linear VDR in the full treatment-by-genotype model, including their pairwise and three-way interactions. RFS used joint site-and-stage stratification; OS used site stratification and stage adjustment. Four-degree-of-freedom partial-likelihood ratio tests compared spline and linear specifications, and two-degree-of-freedom Wald tests jointly assessed the two three-way spline terms. Knots were shared across endpoints and were not optimized against outcomes. All added tests and intervals are nominal and exploratory (Table S6).

Additional post hoc analyses evaluated treatment associations separately within colorectal and gastric cancers using pooled models containing treatment, joint biomarker subgroup, their interaction, age, sex, and pathological stage. Subgroup-specific HRs were obtained from linear combinations of model coefficients. A sensitivity analysis excluded colorectal cancers and used the same covariates, with baseline hazards stratified by the remaining tumor sites. Patient and event counts were tabulated by site, biomarker subgroup, and treatment arm (Table S2). The site-specific and colorectal-exclusion models each contained seven regression coefficients and were interpreted cautiously because of the limited event counts. Stable conventional Cox treatment estimates were not reported for the esophageal CC/VDR Q2–5 subgroup because only one event occurred for each endpoint, with no events in the vitamin D arm. These additional analyses were exploratory; *p*-values and confidence intervals are nominal, and comparisons between site-specific estimates are descriptive rather than formal tests of treatment-effect heterogeneity by site.

Further post hoc analyses separated the two biomarker dimensions within the same 359-patient cohort. For each endpoint, separate single-biomarker models included either FokI genotype (CC versus CT/TT) or VDR expression (Q2–5 versus Q1), treatment, and their interaction. A full model included treatment, genotype, VDR category, all three pairwise interactions, and the three-way treatment-by-genotype-by-VDR interaction. All models adjusted for age, sex, and pathological stage and stratified baseline hazards by primary tumor site. The existing VDR quintile assignments were retained. Treatment HRs for the four biomarker combinations were obtained from linear combinations of full-model coefficients. The three-way term was assessed by a one-degree-of-freedom Wald test. Nested partial-likelihood ratio tests compared the full model (11 coefficients) with each single-biomarker model (7 coefficients), using four degrees of freedom. These comparisons assessed model fit, not discrimination or externally validated predictive performance. All additional *p*-values and confidence intervals are nominal (Table S3).

Absolute risks at 5 years were estimated as one minus the Kaplan–Meier survival function. Within each biomarker subgroup, risk differences were defined as vitamin D minus placebo, equivalent to placebo survival minus vitamin D survival. Approximate 95% Wald confidence intervals used the square root of the sum of the two independent treatment-arm Greenwood variances. These unadjusted, pointwise intervals are nominal and were not corrected for multiplicity. Median follow-up was estimated using reverse Kaplan–Meier analysis of overall survival times, treating original censoring as the event and deaths as censored. Patient counts, events over the full observed follow-up, numbers at risk at 5 years, and follow-up estimates are reported in Table S4.

Exploratory cutoff sensitivity analyses retained the CC-versus-CT/TT genotype classification and evaluated four nested subgroup definitions: FokI CC with tumor VDR expression in Q2–5, Q3–5, Q4–5, or Q5 alone. The Q2–5 definition corresponded to the main analysis. Each subgroup was compared with its corresponding complement within the same 359-patient cohort, with the original VDR quintile assignments retained. For each definition, treatment HRs and 95% CIs for relapse or death were estimated separately within the subgroup and its complement, and treatment-by-subgroup interactions were assessed using the unadjusted pooled models described above. Because the cutoff-defined subgroups were nested and substantially overlapping, these analyses were exploratory and were not considered independent confirmatory tests.

**Figure 1:**
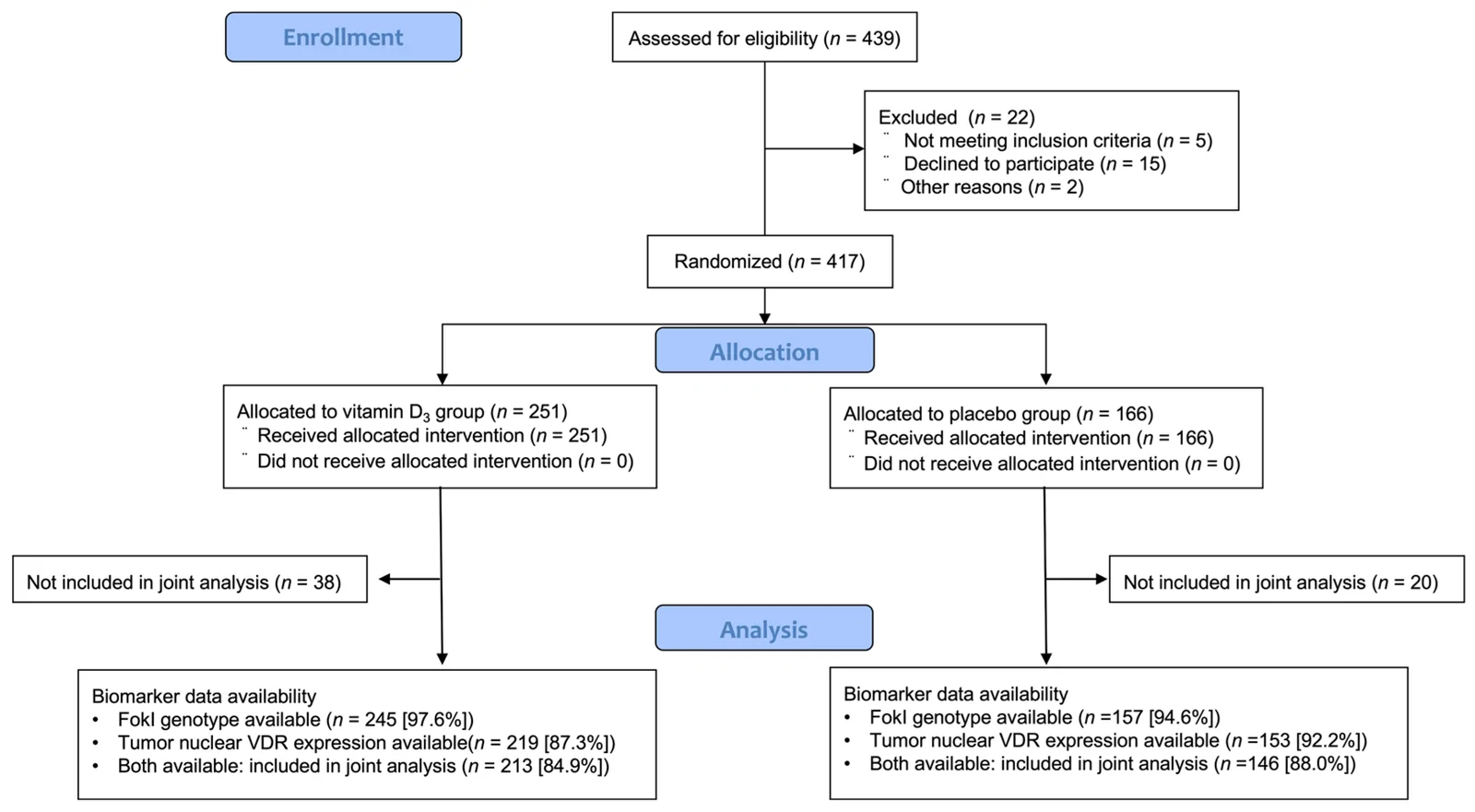
Participant flow and availability of FokI genotype and tumor nuclear VDR expression data for the joint biomarker analysis of the AMATERASU trial.

Participants missing either biomarker were excluded from the joint analyses; missing biomarker values were not imputed. Patients included in each analysis were evaluated according to their original randomized treatment assignment. Biomarker availability and the numbers excluded from the joint analysis are shown in Figure 1.

A post hoc inverse probability weighting (IPW) sensitivity analysis addressed selection into the joint biomarker cohort. A logistic regression model estimated the probability of having both biomarkers available from age, sex, pathological stage, primary tumor site, and randomized treatment assignment. These predictors were observed for all 417 randomized patients. Because both small-intestinal cancer patients had complete biomarker data, this site perfectly predicted inclusion. The logistic model was fitted in the other 415 patients; the two small-intestinal cases were assigned an inclusion probability of 1 as an analysis convention and retained with weight 1. This assignment does not establish their population inclusion probability.

Each of the 359 complete cases received an unstabilized weight equal to the inverse estimated inclusion probability. Probability and weight distributions were inspected, and weighted clinical characteristics were compared descriptively with those of all 417 randomized patients. No weight truncation was applied. Weighted Cox models retained treatment, joint subgroup, their interaction, age, sex, and stage, with baseline hazards stratified by tumor site. Robust sandwich standard errors treated the estimated weights as fixed and did not propagate uncertainty from the selection-model estimation. Missing biomarker values were not imputed. All IPW confidence intervals and *p*-values are nominal. Results and diagnostics are reported in Table S5.

All tests were two-sided. Unless explicitly identified as Bonferroni-corrected, *p*-values and 95% CIs are nominal, and *p* < 0.05 is used as the nominal significance threshold. The original analyses were performed using Stata, version 18.0 (StataCorp LLC, College Station, TX, USA); the additional adjusted analyses were performed using StataNow/SE, version 19.5, with version 18.0 compatibility settings.

Post hoc Bonferroni sensitivity calculations were applied to two explicitly defined sets of unadjusted treatment-by-subgroup interaction tests: the four relapse-free survival tests using the CC/Q2–5, CC/Q3–5, CC/Q4–5, and CC/Q5 definitions; and the two endpoint tests (relapse-free and overall survival) using the main CC/Q2–5 definition. The unadjusted interaction tests considered in these Bonferroni calculations comprise four RFS tests across the VDR cutoff definitions and one OS test for the main classification; the two calculations therefore overlap in the main RFS interaction test. Corrected *p*-values were calculated by multiplying the full-precision nominal *p*-value by the number of tests (four or two), capped at 1; the corresponding significance thresholds were 0.0125 and 0.025. These calculations address multiplicity only within each specified set. Within-subgroup treatment tests and other analyses remain nominal, and confidence intervals were not corrected for multiplicity.

## 3. Results

### 3.1. Study Population

Of the 439 patients assessed for eligibility, 417 were randomized to vitamin D_3_ (*n* = 251) or placebo (*n* = 166). FokI genotype data were available for 402 patients (245 in the vitamin D_3_ group and 157 in the placebo group), and tumor nuclear VDR expression data were available for 372 patients (219 and 153, respectively). Fifty-eight patients lacked data for one or both biomarkers: 13 lacked FokI genotype data only, 43 lacked tumor nuclear VDR expression data only, and 2 lacked both. Consequently, the joint biomarker cohort comprised 359 patients (86.1% of the randomized population), including 213 in the vitamin D_3_ group and 146 in the placebo group (Figure 1).

Among the 15 participants without FokI genotype data, blood samples could not be obtained in seven and separate consent for SNP analysis was not obtained in eight.

FokI genotype availability was therefore 96.4% (402/417); this proportion describes data availability in the randomized cohort rather than an assay-specific technical success rate.

Baseline characteristics of the included and excluded patients are presented in Table S1. Mean age was 66.4 years (SD, 10.6) among included patients and 65.1 years (SD, 10.7) among excluded patients. The proportions of men were 66.0% and 67.2%, and those with stage III disease were 30.1% and 27.6%, respectively. Tumor-site distributions differed descriptively: esophageal cancer accounted for 8.6% versus 15.5%, gastric cancer for 43.2% versus 32.8%, and colorectal cancer for 47.6% versus 51.7%. The proportions assigned to vitamin D were 59.3% and 65.5%, respectively.

In the 359-patient joint biomarker cohort, median follow-up estimated by reverse Kaplan–Meier analysis was 3.93 years (95% CI, 3.77–4.32). Median follow-up was 3.92 years (95% CI, 3.55–4.80) in the CC/VDR Q2–5 subgroup and 3.93 years (95% CI, 3.46–4.34) in the complementary subgroup. Treatment-arm-specific estimates are provided in Table S4.

The joint biomarker analysis included 359 patients with available data for both biomarkers: 213 in the vitamin D_3_ group and 146 in the placebo group. Patients missing either or both biomarkers were excluded from the joint analysis. Percentages were calculated using the number of patients randomized to each treatment group as the denominator. VDR, vitamin D receptor.

### 3.2. Association of FokI Genotype with Relapse or Death

**Figure 2:**
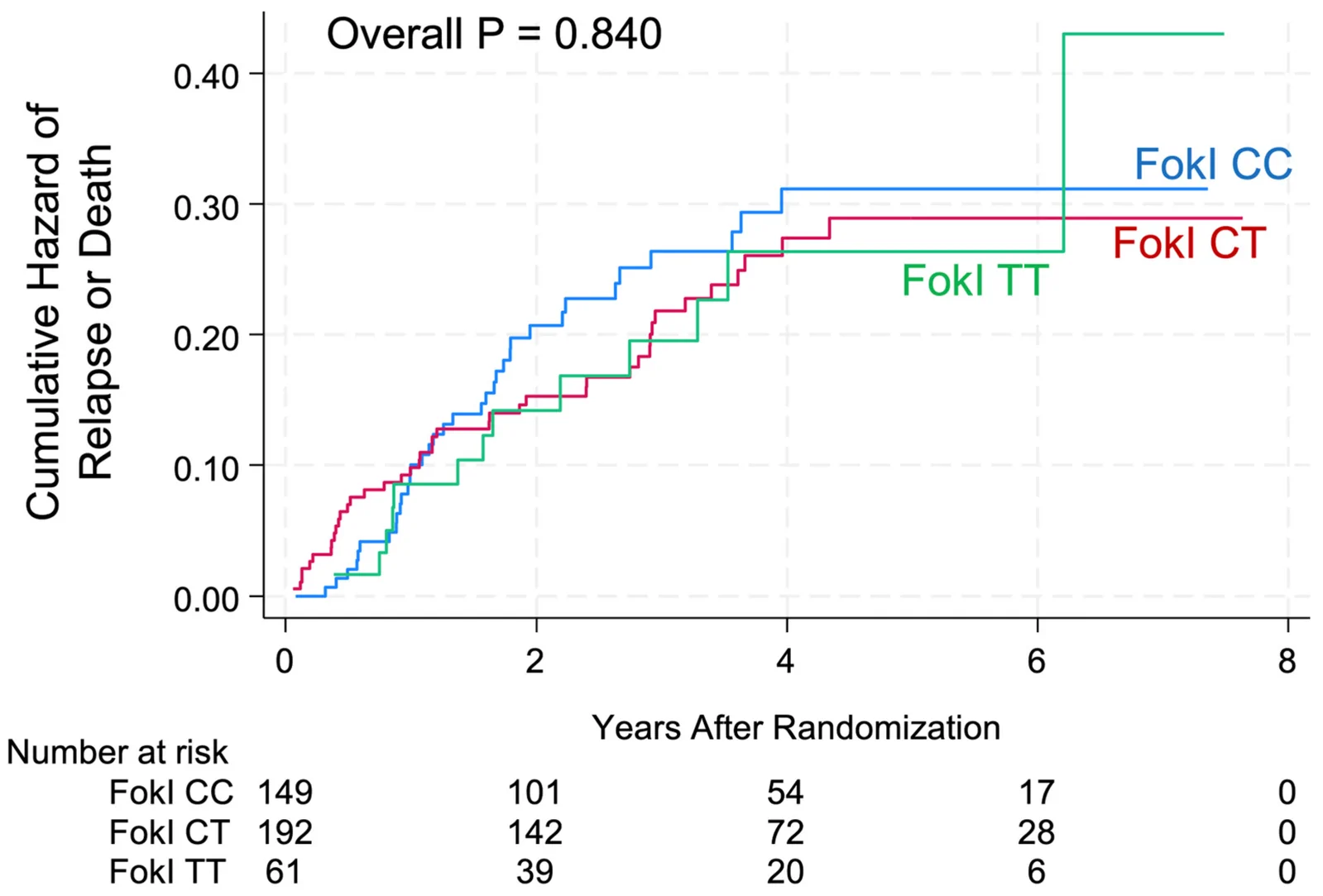
Cumulative hazard of relapse or death according to FokI genotype.

Among the 402 patients with available FokI genotype data, 149 had the CC genotype, 192 had CT, and 61 had TT. During follow-up, 89 patients experienced relapse or death. In an unadjusted Cox proportional hazards model pooling both randomized treatment groups, FokI genotype was not significantly associated with relapse or death (overall likelihood-ratio *p* = 0.840; Figure 2). With TT as the reference genotype, the hazard ratios were 1.12 (95% CI, 0.59–2.11) for CC and 0.98 (95% CI, 0.52–1.82) for CT.

Among the 402 genotyped participants, the C- and T-allele frequencies were 0.60945 and 0.39055. The expected CC, CT, and TT counts under Hardy–Weinberg equilibrium were 149.316, 191.368, and 61.316, respectively, compared with observed counts of 149, 192, and 61. The Pearson chi-square statistic was 0.004382 (1 degree of freedom; *p* = 0.947), providing no evidence of departure from equilibrium in this cohort.

Nelson–Aalen cumulative hazard estimates are shown for patients with the CC (*n* = 149), CT (*n* = 192), and TT (*n* = 61) genotypes, pooling both randomized treatment groups. Numbers below the graph indicate the numbers of patients at risk at the specified time points. The overall association between genotype and relapse or death was evaluated using a likelihood-ratio test in an unadjusted Cox proportional hazards model with genotype entered as a three-level categorical variable (2 degrees of freedom; *p* = 0.840).

### 3.3. Distribution of Tumor Nuclear VDR Expression by Primary Tumor Site

**Figure 3:**
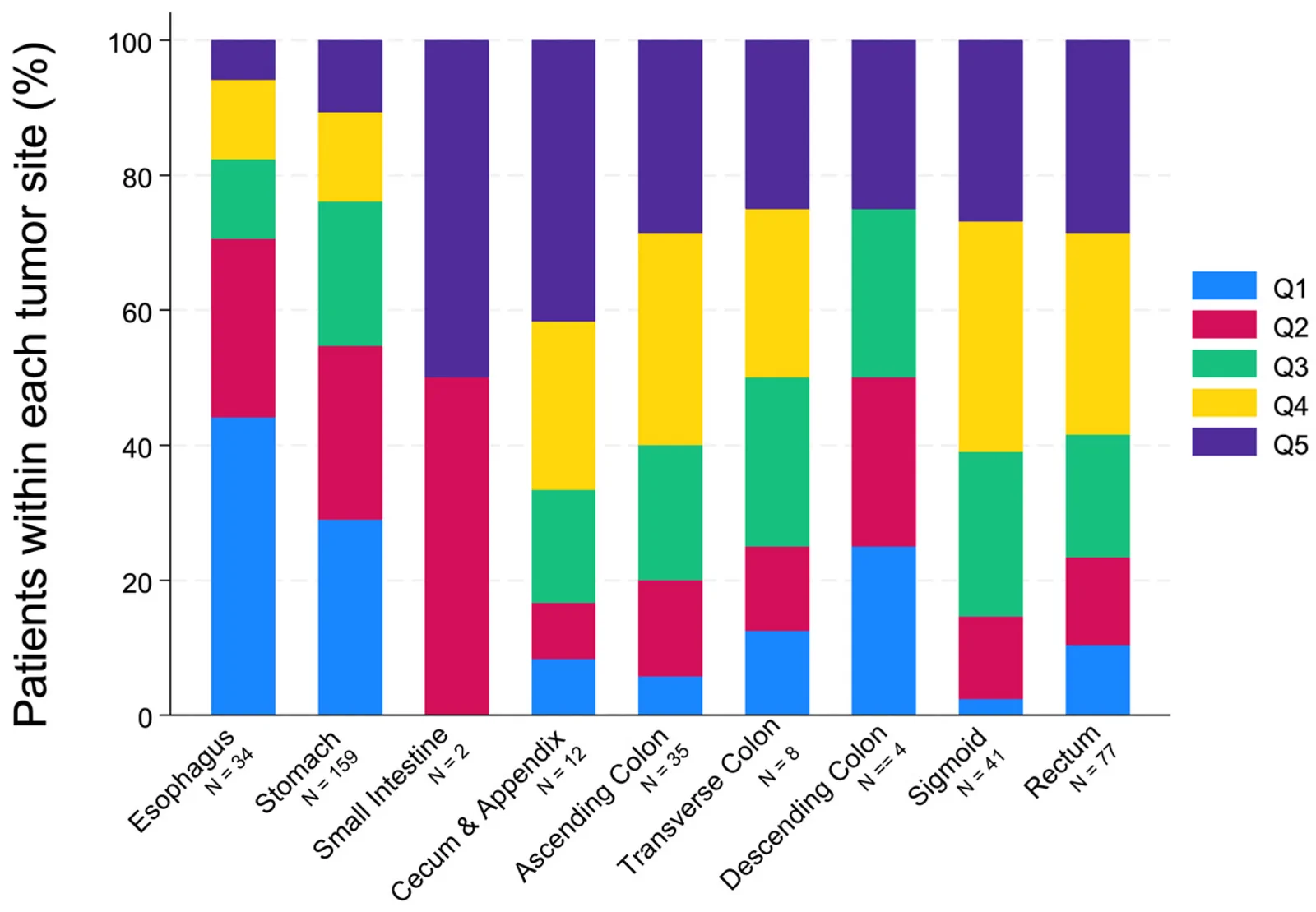
Distribution of tumor nuclear vitamin D receptor expression quintiles by primary tumor site.

Among the 372 patients with available tumor nuclear VDR measurements, the distribution of expression quintiles differed descriptively across primary tumor sites (Figure 3). The lowest quintile (Q1) accounted for 44.1% of esophageal tumors (15/34) and 28.9% of gastric tumors (46/159), compared with 7.9% of colorectal and appendiceal tumors combined (14/177). Conversely, the upper two quintiles (Q4–Q5) accounted for 17.6% (6/34), 23.9% (38/159), and 58.8% (104/177), respectively. Distributions also varied across individual colorectal subsites, with no clear proximal-to-distal gradient apparent on visual inspection. Findings for the small intestine, transverse colon, and descending colon were based on only 2, 8, and 4 patients, respectively, and should be interpreted cautiously.

Stacked bars show the percentages of patients within each anatomical category classified into each tumor nuclear vitamin D receptor (VDR) expression quintile. Quintile cutoffs were determined among all 372 patients with available VDR measurements and applied uniformly across tumor sites; quintiles were not defined separately within each site. Q1 and Q5 denote the lowest and highest expression quintiles, respectively. Sample sizes are indicated below each category. Cecal and appendiceal tumors are grouped together. The distributions are presented descriptively, and categories with small sample sizes should be interpreted cautiously.

### 3.4. Baseline Characteristics by Biomarker-Defined Subgroup and Treatment Assignment

Baseline characteristics of the 359 patients included in the joint biomarker analysis, according to biomarker-defined subgroup and randomized treatment assignment, are summarized in Table 1. The FokI CC and VDR Q2–5 subgroup comprised 110 patients, including 64 assigned to vitamin D_3_ and 46 assigned to placebo. The complementary subgroup comprised 249 patients, including 149 assigned to vitamin D_3_ and 100 assigned to placebo. The distributions of demographic and clinicopathological characteristics are shown in Table 1.

Values are presented as mean (standard deviation) or *n* (%). The FokI CC and VDR Q2–5 subgroup comprised patients with both the CC genotype and tumor nuclear VDR expression in quintiles 2–5. The complementary subgroup comprised all remaining patients with both biomarkers available: those with the CT or TT genotype, VDR expression in quintile 1, or both. Percentages were calculated using the total number of patients in each column as the denominator and may not sum to 100% because of rounding. Continuous variables were summarized using available observations. From left to right, BMI measurements were available for 64, 46, 147, and 100 patients, and baseline 25(OH)D measurements for 62, 46, 148, and 98 patients. VDR expression quintile cutoffs were determined among all 372 patients with available tumor nuclear VDR measurements and applied unchanged to the 359 patients in the joint analysis. Q1 and Q5 denote the lowest and highest expression quintiles, respectively. BMI, body mass index; VDR, vitamin D receptor; 25(OH)D, 25-hydroxyvitamin D; Q, quintile.

### 3.5. Unadjusted Vitamin D Supplementation Associations by Jointly Defined FokI–VDR Subgroup

**Figure 4:**
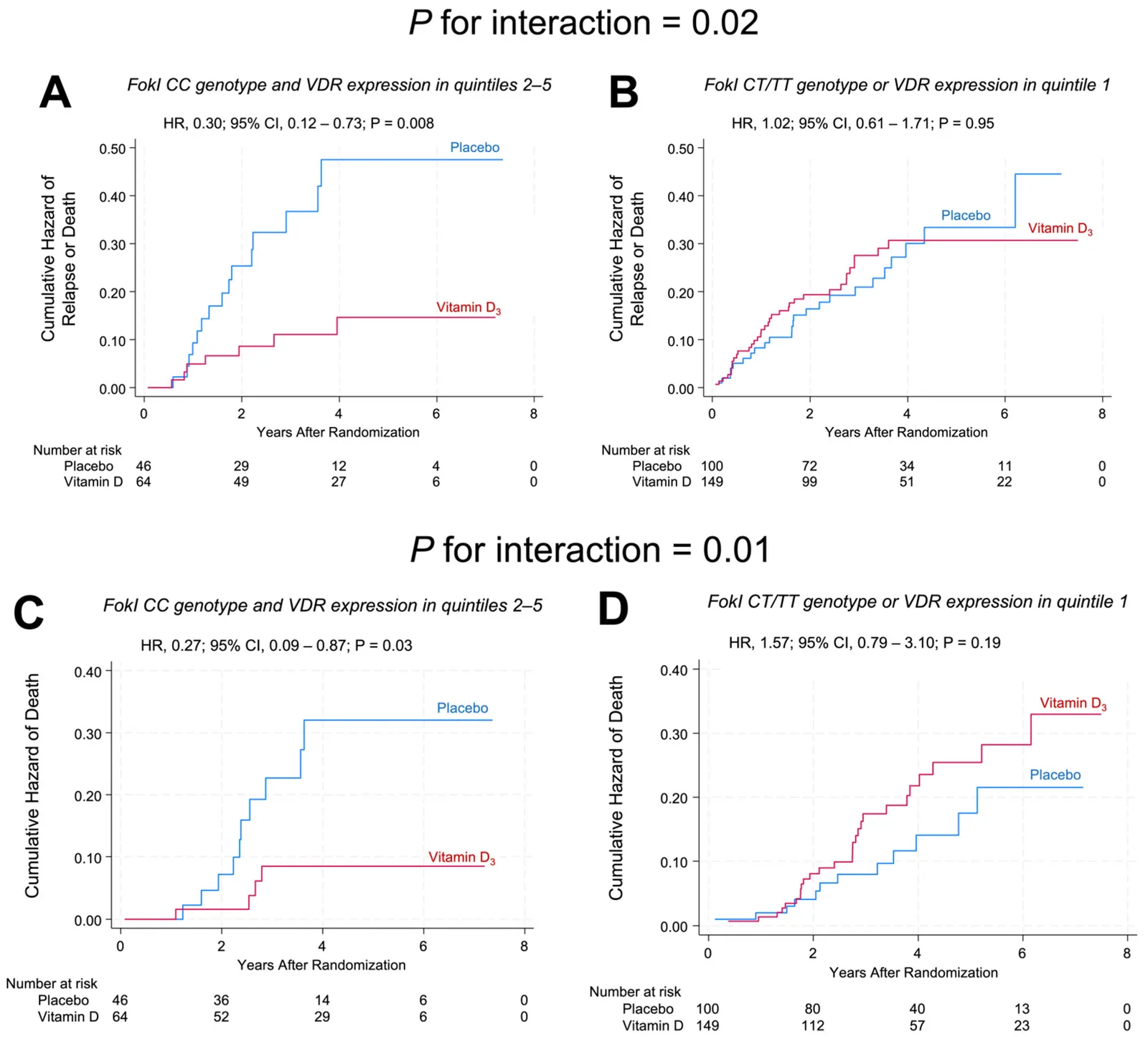
Unadjusted associations between vitamin D_3_ supplementation and clinical outcomes according to jointly defined FokI genotype and tumor nuclear VDR expression subgroups. Nelson–Aalen cumulative hazard curves compare vitamin D_3_ supplementation with placebo. (**A**) Relapse or death in patients with the FokI CC genotype and tumor nuclear vitamin D receptor (VDR) expression in quintiles 2–5 (*n* = 110; vitamin D_3_, *n* = 64; placebo, *n* = 46). (**B**) Relapse or death in the complementary subgroup (*n* = 249; vitamin D_3_, *n* = 149; placebo, *n* = 100). (**C**) All-cause mortality in patients with the FokI CC genotype and tumor nuclear VDR expression in quintiles 2–5 (*n* = 110; vitamin D_3_, *n* = 64; placebo, *n* = 46). (**D**) All-cause mortality in the complementary subgroup (*n* = 249; vitamin D_3_, *n* = 149; placebo, *n* = 100).

Among the 359 patients included in the joint biomarker analysis, 81 experienced relapse or death. In the FokI CC and VDR Q2–5 subgroup, events occurred in 7 of 64 vitamin D patients and 15 of 46 placebo patients (22 events in 110 patients); corresponding counts in the complementary subgroup were 35 of 149 and 24 of 100 (59 events in 249 patients). In the CC/VDR Q2–5 subgroup, vitamin D supplementation was associated in an unadjusted model with a lower hazard of relapse or death than placebo (HR, 0.30; 95% CI, 0.12–0.73; *p* = 0.008; Figure 4A). Estimated 5-year relapse-free survival was 86.25% with vitamin D and 61.64% with placebo. The absolute risk difference for relapse or death at 5 years was −24.61 percentage points (approximate 95% CI, −43.37 to −5.85). In the complementary subgroup, the unadjusted HR was 1.02 (95% CI, 0.61–1.71; *p* = 0.95; Figure 4B), 5-year relapse-free survival was 73.45% and 71.40%, respectively, and the risk difference was −2.05 percentage points (95% CI, −15.30 to 11.20). The treatment-by-subgroup interaction was nominally significant (*p* = 0.02).

For all-cause mortality, 4 of 64 vitamin D patients and 10 of 46 placebo patients died in the CC/VDR Q2–5 subgroup (14 deaths); corresponding counts in the complementary subgroup were 27 of 149 and 12 of 100 (39 deaths). In the CC/VDR Q2–5 subgroup, the unadjusted treatment HR was 0.27 (95% CI, 0.09–0.87; *p* = 0.03; Figure 4C). Estimated 5-year overall survival was 91.78% with vitamin D and 72.20% with placebo, with a death risk difference of −19.57 percentage points (approximate 95% CI, −36.53 to −2.62). In the complementary subgroup, the unadjusted HR was 1.57 (95% CI, 0.79–3.10; *p* = 0.19; Figure 4D), 5-year overall survival was 77.43% and 83.78%, respectively, and the death risk difference was 6.35 percentage points (95% CI, −6.31 to 19.02). The treatment-by-subgroup interaction was nominally significant (*p* = 0.01). Table S4 presents these unadjusted estimates; all absolute risk-difference intervals are nominal. At 5 years, the RFS risk sets included 17 vitamin D and 8 placebo patients in the CC/VDR Q2–5 subgroup and 33 and 20 in the complement; corresponding OS numbers were 19 and 10, and 36 and 26.

Nelson–Aalen cumulative hazard curves compare vitamin D_3_ supplementation with placebo for relapse or death (A, B) and all-cause mortality (C, D). Panels A and C show patients with the FokI CC genotype and tumor nuclear vitamin D receptor (VDR) expression in quintiles 2–5 (*n* = 110; vitamin D_3_, *n* = 64; placebo, *n* = 46). Panels B and D show the complementary subgroup, comprising patients with the CT or TT genotype, VDR expression in quintile 1, or both (*n* = 249; vitamin D_3_, *n* = 149; placebo, *n* = 100). VDR expression quintile cutoffs were determined among all 372 patients with available tumor nuclear VDR measurements and applied unchanged to the joint biomarker cohort. Unadjusted hazard ratios (HRs) and 95% confidence intervals (CIs) were estimated using separate Cox proportional hazards models within each subgroup, with randomized treatment assignment as the sole covariate. Unadjusted interaction *p*-values were obtained from one-degree-of-freedom Wald tests in models containing treatment assignment, subgroup membership, and their interaction. Numbers below each panel indicate the numbers of patients at risk at the specified time points. All *p*-values are two-sided and nominal, without adjustment for multiple testing.

### 3.6. Adjusted Analyses and Model Assumptions

All 359 patients had complete data for age, sex, pathological stage, and primary tumor site; 81 experienced relapse or death and 53 died. In pooled models adjusted for age, sex, and stage and stratified by tumor site, the treatment HR in the FokI CC and VDR Q2–5 subgroup was 0.44 (95% CI, 0.18–1.10; *p* = 0.078) for relapse or death and 0.46 (95% CI, 0.14–1.51; *p* = 0.199) for all-cause mortality. The corresponding treatment HRs in the complementary subgroup were 0.87 (95% CI, 0.50–1.50; *p* = 0.620) and 1.33 (95% CI, 0.65–2.70; *p* = 0.436). Adjusted treatment-by-subgroup interaction *p*-values were 0.211 and 0.135, respectively. Thus, the adjusted analyses did not provide statistically clear evidence of a treatment effect within the CC/VDR Q2–5 subgroup or of treatment-effect heterogeneity between the subgroups.

Global Schoenfeld residual tests yielded *p* = 0.225 for relapse-free survival and *p* = 0.414 for overall survival. The stage III term in the relapse-free survival model showed a possible departure from proportional hazards (*p* = 0.014). In the sensitivity analysis stratified jointly by tumor site and stage, the treatment HR for relapse or death in the FokI CC and VDR Q2–5 subgroup was 0.45 (95% CI, 0.18–1.14; *p* = 0.091), with a treatment-by-subgroup interaction *p*-value of 0.246. This analysis yielded a similar interpretation without imposing proportional hazards for stage.

### 3.7. Sensitivity to VDR Expression Cutoffs

**Figure 5:**
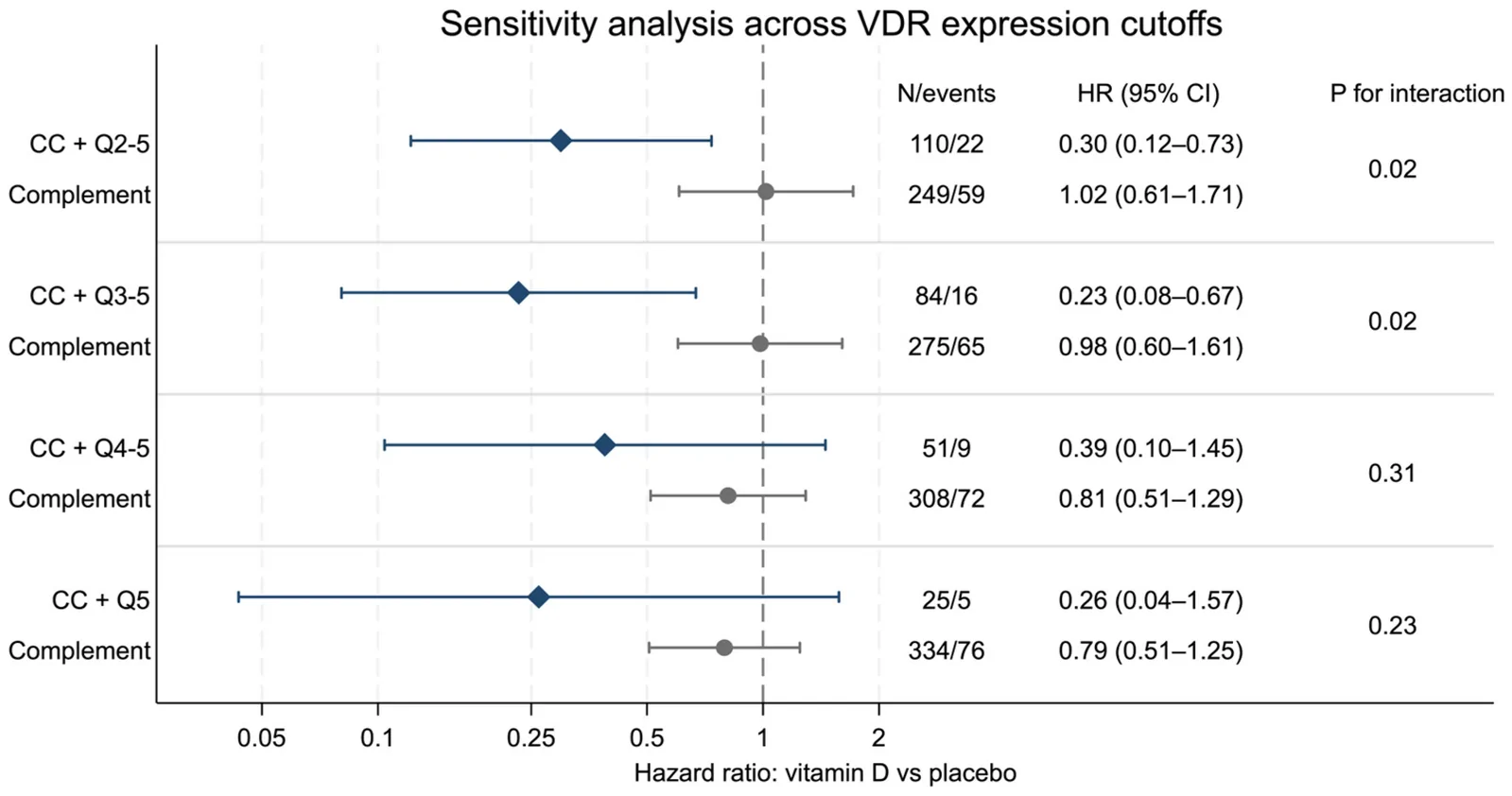
Unadjusted sensitivity analysis of the effect of vitamin D_3_ supplementation on relapse or death across tumor nuclear VDR expression cutoffs.

Across the four nested subgroup definitions, unadjusted treatment HR point estimates for relapse or death in the jointly defined CC subgroups were below 1, although confidence intervals and interaction *p*-values varied (Figure 5). The nominal interaction *p*-value was 0.02 for the Q2–5 definition used in the main analysis. When the definition was restricted to CC and Q3–5, the subgroup included 84 patients with 16 events; the treatment HR was 0.23 (95% CI, 0.08–0.67), compared with 0.98 (95% CI, 0.60–1.61) in the corresponding complementary subgroup (nominal *p* for interaction = 0.02). Further restriction to CC and Q4–5 or CC and Q5 yielded 51 patients with 9 events and 25 patients with 5 events, respectively. The corresponding treatment HRs were 0.39 (95% CI, 0.10–1.45) and 0.26 (95% CI, 0.04–1.57), with nominal interaction *p*-values of 0.31 and 0.23.

The interpretation of the unadjusted interaction findings depended on the scope of the post hoc multiplicity correction. For the four relapse-free survival cutoff interaction tests, Bonferroni-corrected *p*-values were 0.0870, 0.0647, 1.0000, and 0.9316 for the Q2–5, Q3–5, Q4–5, and Q5 definitions, respectively; none was below 0.05. For transparency, the separate calculation restricted to the two endpoint interaction tests for the main CC/Q2–5 definition yielded corrected *p*-values of 0.0435 for relapse-free survival and 0.0196 for overall survival. These limited-scope results were not based on a prespecified testing hierarchy and do not provide correction across the full set of exploratory analyses.

The forest plot shows unadjusted HRs and 95% CIs for relapse or death comparing vitamin D_3_ supplementation with placebo. Diamonds represent the FokI CC subgroups defined by the indicated tumor nuclear vitamin D receptor (VDR) expression quintiles; circles represent the corresponding complementary subgroups. N/events indicates the number of patients and relapse-or-death events in each subgroup, with both treatment groups combined. The horizontal axis uses a logarithmic scale, and the vertical dashed line indicates an HR of 1.

### 3.8. Analyses by Primary Tumor Site

Among the 110 patients in the CC/VDR Q2–5 subgroup, 64 had colorectal cancer, 39 gastric cancer, and 7 esophageal cancer; colorectal cancers accounted for 15 of the 22 relapse-or-death events and 8 of the 14 deaths in this subgroup. Site- and treatment-specific counts and estimates are presented in Table S2. Within colorectal cancer (171 patients; 40 relapse-or-death events and 23 deaths), the adjusted treatment HRs in the CC/VDR Q2–5 subgroup were 0.42 (95% CI, 0.14–1.24; *p* = 0.116) for relapse-free survival and 0.36 (95% CI, 0.07–1.86; *p* = 0.224) for overall survival. The corresponding treatment-by-biomarker-subgroup interaction *p*-values were 0.276 and 0.126. Within gastric cancer (155 patients; 26 relapse-or-death events and 19 deaths), adjusted treatment HRs were 0.91 (95% CI, 0.16–5.27; *p* = 0.916) and 1.23 (95% CI, 0.19–8.02; *p* = 0.832), with interaction *p*-values of 0.802 and 0.829, respectively.

After excluding colorectal cancers, 188 patients remained, with 41 relapse-or-death events and 30 deaths. The adjusted treatment HRs in the CC/VDR Q2–5 subgroup were 0.41 (95% CI, 0.07–2.24; *p* = 0.302) for relapse-free survival and 0.59 (95% CI, 0.10–3.53; *p* = 0.563) for overall survival; interaction *p*-values were 0.402 and 0.518, respectively. The two patients with small-intestinal cancer had no events and contributed no information to coefficient estimation through the partial likelihood in these site-stratified models. For esophageal cancer, the CC/VDR Q2–5 subgroup comprised only 7 patients: the placebo arm had one relapse-or-death event and one death among 4 patients, and the vitamin D arm had no events among 3 patients. Stable finite treatment HRs were not reported for this subgroup.

### 3.9. Separate and Combined Biomarker Analyses

The four biomarker groups comprised 49 patients with CT/TT and VDR Q1 (15 relapse-or-death events; 12 deaths), 176 with CT/TT and Q2–5 (36 events; 19 deaths), 24 with CC and Q1 (8 events; 8 deaths), and 110 with CC and Q2–5 (22 events; 14 deaths). Treatment-specific counts and estimates are presented in Table S3. In the adjusted single-biomarker models, treatment-by-genotype interaction *p*-values were 0.618 for relapse-free survival and 0.367 for overall survival; treatment-by-VDR interaction *p*-values were 0.767 and 0.942, respectively. In the full models, three-way interaction *p*-values were 0.270 and 0.347, respectively.

Full-model adjusted treatment HRs for relapse-free survival were 0.65 (95% CI, 0.22–1.96) for CT/TT with VDR Q1, 0.86 (0.43–1.68) for CT/TT with Q2–5, 1.50 (0.18–12.76) for CC with Q1, and 0.44 (0.18–1.10) for CC with Q2–5. Corresponding overall survival HRs were 0.83 (0.23–2.94), 1.41 (0.54–3.64), 1.13 (0.13–10.27), and 0.46 (0.14–1.51), respectively. All confidence intervals included 1.

For relapse-free survival, the full model did not show a statistically clear improvement in fit over the genotype-only model (likelihood-ratio chi-square, 2.96; 4 degrees of freedom; *p* = 0.565) or the VDR-only model (chi-square, 2.04; 4 degrees of freedom; *p* = 0.728). Corresponding overall survival comparisons yielded chi-square values of 4.49 (*p* = 0.344) and 3.16 (*p* = 0.532), respectively, each with 4 degrees of freedom. These exploratory comparisons did not demonstrate improved model fit for the combined specification.

### 3.10. Sensitivity Analysis for Missing Biomarker Data

Estimated inclusion probabilities ranged from 0.6663 to 1.0000 across the 417 randomized patients, including the two small-intestinal cases assigned probability 1. Among the 359 complete cases, IPW weights ranged from 1.0000 to 1.5009 (mean, 1.1616; median, 1.1458), with a total weight of 417.03. Weighted mean age was 66.25 years, compared with 66.23 years in the full randomized cohort. Weighted proportions for sex, stage, tumor site, and treatment assignment were descriptively close to the full-cohort proportions (Table S5).

In the weighted, clinicopathologically adjusted models, the treatment HR in the CC/VDR Q2–5 subgroup was 0.43 (95% CI, 0.16–1.12; *p* = 0.085) for relapse-free survival and 0.44 (95% CI, 0.12–1.59; *p* = 0.211) for overall survival. Corresponding treatment-by-subgroup interaction *p*-values were 0.2020 and 0.1349. These estimates were close to the unweighted adjusted estimates. The analyses used 359 actual patients with 81 relapse-or-death events and 53 deaths; weighted totals do not represent additional observed patients or events.

### 3.11. Continuous VDR Analyses and Model Diagnostics

In the continuous VDR models, three-way interaction *p*-values were 0.2290 for RFS and 0.2122 for OS. Global proportional hazards test *p*-values were 0.5164 and 0.5068, respectively. The RFS stage III term showed a possible departure (*p* = 0.0233); joint site-and-stage stratification gave a global *p* of 0.8858 and a three-way interaction *p* of 0.244. In this RFS sensitivity model, the ratio of treatment HRs per 100-point increase in VDR was 0.73 (95% CI, 0.33–1.62; *p* = 0.442) in CC and 1.32 (0.74–2.37; *p* = 0.350) in CT/TT. Corresponding OS ratios were 0.63 (0.20–1.97; *p* = 0.424) and 1.53 (0.68–3.43; *p* = 0.299). Joint treatment-by-VDR tests yielded *p* = 0.4859 for RFS and *p* = 0.4261 for OS. These ratios describe changes in treatment HR with VDR, not treatment HRs themselves.

The OS three-way term showed a possible proportional hazards departure (*p* = 0.0461), despite the nonsignificant global test. The added log-time interaction was not statistically significant (Wald *p* = 0.649; likelihood-ratio *p* = 0.6749); this specific alternative does not exclude other time patterns. Maximum absolute standardized DFBETAs for the three-way term were 0.312 for RFS and 0.374 for OS. Omitting the respective most influential case changed the exponentiated three-way coefficient from 0.554 (95% CI, 0.204–1.499; *p* = 0.244) to 0.461 (0.164–1.295; *p* = 0.142) for RFS, and from 0.409 (0.101–1.665; *p* = 0.2122) to 0.280 (0.058–1.357; *p* = 0.114) for OS. Estimates shifted and remained imprecise; these findings do not establish the absence of influential observations.

Spline models did not demonstrate improved fit over linear VDR models (RFS: likelihood-ratio chi-square = 2.44, 4 df, *p* = 0.6547; OS: chi-square = 0.94, 4 df, *p* = 0.9182). Joint three-way spline interaction *p*-values were 0.6531 and 0.6288, respectively. These results neither establish linearity nor exclude nonlinear effect modification (Table S6).

## 4. Discussion

### 4.1. Principal Findings

In this exploratory post hoc analysis of the AMATERASU randomized trial, unadjusted analyses suggested a lower hazard of relapse or death with vitamin D_3_ supplementation among patients with both the FokI CC genotype and tumor nuclear VDR expression in quintiles 2–5, with a nominally significant treatment-by-subgroup interaction. A similar unadjusted pattern was observed for all-cause mortality. However, after accounting for age, sex, pathological stage, and primary tumor site, the treatment HR estimates moved toward 1 and their confidence intervals included 1; the treatment-by-subgroup interactions were also no longer nominally significant. The relapse-free survival sensitivity analysis allowing separate baseline hazards for each tumor-site and stage combination gave a similar interpretation. Together with the absence of a statistically significant overall benefit in the parent trial [13], these results do not establish differential supplementation benefit according to this joint classification.

The proposed classification combines a genotype-based proxy of receptor functional potential with tumor receptor abundance. Across the four VDR cutoffs, unadjusted treatment HR point estimates in the jointly defined CC subgroups were below 1, but treatment-by-subgroup interactions were not consistently nominally significant and estimates became less precise with more restrictive definitions. Given the nested, overlapping subgroups, multiple exploratory comparisons, absence of a prespecified multiplicity strategy, and attenuation after clinicopathological adjustment, the findings remain hypothesis-generating. They neither establish an optimal VDR threshold nor justify clinical treatment selection, but support independent validation in adequately powered randomized studies using a prospectively specified biomarker definition.

### 4.2. Prognostic Relevance and Potential Treatment-Effect Modification

FokI genotype alone was not significantly associated with relapse or death in our cohort. This finding is compatible with the uncertain prognostic relevance of VDR polymorphisms summarized by Thet et al. [22] and the absence of a detected association between FokI and survival outcomes in the colorectal cancer cohort reported by Zhu et al. [23]. However, a biomarker’s association with prognosis and its ability to identify differences in treatment benefit are distinct questions [24]. The absence of a statistically significant prognostic association therefore neither establishes nor excludes a potential role for FokI in modifying the effect of vitamin D supplementation.

The nominal treatment-by-subgroup interactions in the unadjusted analyses were not retained after clinicopathological adjustment. The additional single-biomarker and full models did not provide statistically clear evidence of genotype-by-treatment, VDR-by-treatment, or three-way interactions. The full model also did not demonstrate improved fit over either single-biomarker model. These results do not establish the joint classification as a superior predictor, identify which biomarker accounts for the original pooled association, or demonstrate a biological interaction between genotype and tumor expression. A nonsignificant three-way term does not establish that all four treatment effects are equal. The estimates remain imprecise, and model fit comparisons do not constitute an assessment of discrimination or independent predictive validation.

### 4.3. Biological Rationale for the Joint Biomarker Classification

The joint classification was motivated by the possibility that vitamin D responsiveness depends on both receptor function and receptor abundance. The shorter, C-allele-associated VDR isoform has shown greater transcriptional activity and more efficient interaction with transcription factor IIB in experimental studies [15,16], and greater sensitivity to the growth-inhibitory effects of active vitamin D has been observed in engineered breast cancer cells [17]. However, the functional consequences of FokI variation differ across experimental systems and signaling pathways [18,19]. The CC genotype should therefore be regarded as a proxy for potential functional differences, not as a direct measure of enhanced VDR activity in an individual tumor.

Receptor abundance provides a complementary rationale. In colon cancer models, active vitamin D promotes epithelial differentiation through VDR-dependent mechanisms [4], and SNAIL-mediated repression of VDR expression reduces vitamin D responsiveness and the antitumor activity of a vitamin D analog in a xenograft model [25]. These findings suggest that low receptor abundance may constrain direct tumor responses, but do not establish that VDR expression in Q2–5 is biologically sufficient or that responses to active vitamin D or its analogs translate directly to benefit from oral vitamin D_3_ supplementation.

The present analysis did not measure receptor transcriptional activity or downstream signaling. Tumor nuclear VDR expression also does not capture potential effects mediated by stromal fibroblasts or host immune mechanisms [5,6,18]. The biological rationale remains plausible, but the unadjusted clinical pattern and its attenuation after adjustment do not demonstrate the proposed mechanism. Mechanistic studies linking genotype and receptor expression to functional responses are needed.

### 4.4. Interpretation of VDR Expression Cutoff Sensitivity Analyses

The interpretation of the unadjusted interaction findings was sensitive to the multiplicity adjustment framework. The Q2–5 and Q3–5 interactions did not remain significant after Bonferroni correction across four cutoff tests (corrected *p* = 0.0870 and 0.0647). Although some post hoc limited-scope corrections yielded *p*-values below 0.05, they lacked a prespecified testing hierarchy and did not control multiplicity across the full set of exploratory analyses. Moreover, clinicopathologically adjusted interactions were not statistically significant. The findings should therefore be considered hypothesis-generating rather than confirmatory. The nested, overlapping cutoff analyses do not constitute independent replication. More restrictive definitions yielded fewer patients and events, wider intervals, and nonsignificant interactions, while changing the complementary subgroup. These findings do not identify an optimal threshold. Categorizing a continuous biomarker can discard information and make results cutoff-dependent [26]; independent validation using a prospectively specified definition is required before treatment selection [27].

### 4.5. Limitations and Clinical Implications

Several limitations should be considered. This was an exploratory post hoc analysis of a trial that did not demonstrate a statistically significant improvement in its primary outcome in the overall population [13]. The joint biomarker hypothesis was not prospectively specified, and the multiple analyses increase the likelihood of chance findings. Both biomarkers were available for 359 of the 417 randomized patients; restricting the analysis to these patients may have introduced selection bias if biomarker availability was related to patient characteristics or outcomes. The limited number of events in the FokI CC and VDR Q2–5 subgroup reduces precision and permits chance baseline imbalances within the randomized subgroups. In particular, within the CC/VDR Q2–5 subgroup, stage III disease was present in 21.9% of the vitamin D group and 41.3% of the placebo group (Table 1), an imbalance that could favor the unadjusted treatment estimate. The treatment HRs moved toward 1 after simultaneous adjustment for clinicopathological factors, although this change cannot be attributed solely to stage. The adjusted models were also post hoc, assumed common covariate effects across biomarker subgroups, and remained imprecise; the CC/VDR Q2–5 subgroup had only 22 relapse-or-death events and 14 deaths. Wide confidence intervals preclude concluding either that supplementation has no effect or that the treatment effects are equivalent between subgroups.

Table 1 also shows differences in mean age between the vitamin D and placebo arms: 67.8 versus 64.5 years in CC/VDR Q2–5 and 67.9 versus 64.2 years in the complement. Male proportions were 57.8% versus 60.9% and 73.8% versus 62.0%, respectively. Within CC/VDR Q2–5, colorectal cancers accounted for 60.9% versus 54.3% of the treatment arms, compared with 43.0% in both complementary-subgroup arms. These descriptive imbalances need not favor the same arm, and tumor-site differences may accompany differences in stage, receptor expression, and prognosis. Adjustment reduces confounding by the specified measured factors but does not establish causal subgroup effects or overcome limited precision.

Colorectal cancers contributed most patients and events within the CC/VDR Q2–5 subgroup. However, the adjusted site-specific and colorectal-exclusion estimates were imprecise and did not establish treatment-effect heterogeneity between biomarker subgroups. The data therefore cannot establish that the pooled finding was driven exclusively by colorectal cancers, nor demonstrate benefit outside colorectal cancer. Similar point estimates in the colorectal and colorectal-exclusion analyses do not establish equivalence across sites. Sparse events, particularly in gastric and esophageal cancers, and seven regression coefficients in each adjusted model limit stability and raise concerns about overfitting. These analyses do not identify which tumor site accounts for the observed pooled association.

The full biomarker models estimated 11 regression coefficients from 81 relapse-or-death events or 53 deaths. The CC/VDR Q1 cell contained only 24 patients, including 6 placebo patients with one event for each endpoint. Sparse cells and limited events may produce unstable coefficients, wide confidence intervals, and overfitting, particularly for the three-way interaction. Nonsignificant interaction and model-comparison tests do not establish the absence of effect modification or equivalence between models. These analyses remain exploratory and require independent validation.

Continuous and spline VDR analyses did not provide statistically clear evidence of the tested effect modification, but limited precision prevents concluding that modification is absent. The spline models estimated 13 coefficients from 81 RFS events and 15 coefficients from 53 deaths, increasing the risk of overfitting. Several Schoenfeld tests were performed; the nominal OS three-way departure and nonsignificant targeted log-time test leave uncertainty about proportional hazards. Influence diagnostics focused on the three-way coefficient, and one-case deletion changed its magnitude without yielding a statistically clear interaction. These checks do not establish model adequacy for all parameters. Neither nonsignificant nonlinearity tests nor this limited influence assessment validates the original VDR cutoff or removes the uncertainty from exploratory threshold selection.

Only 22 relapse-or-death events and 14 deaths occurred in the CC/VDR Q2–5 subgroup. The large unadjusted relative and absolute associations may therefore reflect chance or subgroup imbalances and should be interpreted alongside the attenuated, imprecise adjusted estimates. Median follow-up was 3.93 years, and only 8–36 patients per treatment-by-biomarker group remained at risk at 5 years, limiting precision of the 5-year estimates. The approximate risk-difference confidence intervals rely on large-sample methods despite sparse events; they are unadjusted and nominal and do not establish confirmatory treatment benefit. In the complementary subgroup, the mortality HR above 1 and the positive 5-year death risk difference are compatible with potential harm, although chance remains a possible explanation. Their nonsignificance does not exclude clinically important harm. These exploratory survival analyses do not establish safety and provide no basis for changing clinical practice.

The included and excluded patients had broadly similar age, sex, and stage distributions descriptively, but their tumor-site composition and treatment allocation proportions differed (Table S1). The IPW sensitivity analysis produced estimates close to the unweighted adjusted analyses, but cannot rule out selection bias. It relies on adequate modeling of biomarker availability and on the observed clinical factors sufficiently accounting for selection relevant to the outcome and biomarker classification. Selection related to unobserved biomarker values or other unmeasured factors remains possible. The probability-1 convention for only two small-intestinal cases is uncertain; these patients had no events for either endpoint and contributed no partial-likelihood information to coefficient estimation in the site-stratified Cox models. The robust intervals also treat estimated weights as fixed, omitting selection-model estimation uncertainty. These limitations preclude claiming that weighting eliminated selection bias.

Nominal *p*-values from multiple subgroup, endpoint, and cutoff analyses should not be overinterpreted as confirmatory evidence. The interpretation of the unadjusted interaction findings depended on the multiplicity adjustment framework. Results below 0.05 under some post hoc limited-scope corrections do not establish a robust treatment-by-biomarker association. The two overlapping Bonferroni test sets were defined post hoc; neither constitutes a prespecified testing hierarchy nor controls multiplicity across the full set of exploratory analyses. Within-subgroup treatment findings and confidence intervals also remain subject to multiplicity. Together with the nonsignificant clinicopathologically adjusted interaction tests, these limitations support a hypothesis-generating interpretation and require prospective validation with a prespecified analysis and multiplicity strategy.

Biomarker measurement also has limitations. VDR staining did not include positive or negative controls. The previously reported Spearman correlation of 0.83 between pathologists’ scores [21] does not establish absolute interobserver agreement or reproducibility of the biomarker classification. ICC and categorical agreement measures were not available, and averaging scores does not resolve this limitation. Staining specificity was not independently established through controls. Further genotyping quality-control and personnel-blinding details could not be confirmed; the absence of a detected Hardy–Weinberg departure does not establish genotyping accuracy. Independent validation requires appropriate staining controls, documented genotyping quality control, and formal assessment of scoring and classification reproducibility.

The biomarker definition also requires further validation. VDR quintile boundaries were derived from the distribution among the 372 patients with available measurements and may not transfer directly to populations with different tumor-site distributions or assay conditions. External validation should use a standardized assay, reproducible scoring, and a fixed cutoff specified before outcome analysis; redefining quintiles within each validation cohort would evaluate a different classification. The inclusion of several digestive tract cancer sites and recruitment at a single center in Japan also limit generalizability. Archived specimens from independent randomized trials could support an initial validation if the assay, classification, and analysis plan are specified in advance [28].

Generalizability is also limited by genetic and physiological variation in the wider vitamin D pathway. Jones et al. reported population differences in variants across loci involved in vitamin D synthesis, transport, metabolism, and signaling, including DHCR7/NADSYN1, GC, CYP2R1, CYP24A1, and VDR [29]. Thus, external validity involves more than differences in FokI allele frequencies alone. An observational comparison of Greenlanders and Danes also found differences in vitamin D metabolites and calcium-regulating hormones, alongside associations with diet and season [30]. These findings motivate consideration of genetic background and environmental context when evaluating supplementation, but do not establish differences in cancer treatment response.

The present analysis did not evaluate interactions of FokI and tumor VDR expression with the wider vitamin D pathway or determine whether the biomarker association differs across populations. Hypothesized adaptations in calcium handling, vitamin D activation or transport, and receptor function may be relevant, but were not tested here. Neither enhanced VDR ligand binding nor a lower homeostatic set-point or increased susceptibility to supplementation-induced hypervitaminosis can be inferred from these data. Similar skin pigmentation or UVB exposure does not establish equivalent metabolic responses, and population labels cannot determine an individual’s response. Validation in diverse populations should assess the joint biomarker alongside relevant genetic, metabolic, dietary, and UVB-related factors, and evaluate both benefits and harms before clinical use.

The present findings do not justify using FokI genotype and tumor nuclear VDR expression to recommend or withhold vitamin D supplementation for reducing cancer recurrence or mortality. Their clinical relevance lies in identifying a candidate classification for further testing. A prospective validation trial should include both biomarker-defined subgroups, randomize treatment within each subgroup, and be powered to evaluate the treatment-by-subgroup interaction [31]. It should also assess whether the joint classification adds predictive information beyond either biomarker alone and established clinicopathological characteristics, and quantify absolute benefits and harms before clinical implementation [32].

## 5. Conclusions

In this exploratory post hoc analysis of the AMATERASU randomized trial, unadjusted associations suggesting benefit among patients with the FokI CC genotype and tumor nuclear VDR expression in Q2–5 were attenuated after accounting for age, sex, pathological stage, and primary tumor site. Neither the adjusted within-subgroup treatment effects nor the treatment-by-subgroup interactions were statistically significant. The findings do not establish the joint classification as a predictive biomarker and do not support clinical treatment selection; independent validation is required.

## Figures and Tables

**Table 1:** Baseline characteristics of the joint biomarker cohort by biomarker-defined subgroup and randomized treatment assignment.

	FokI CC and VDR Q2–5		Complementary Subgroup	
	Vitamin D_3_ (*n* = 64)	Placebo (*n* = 46)	Vitamin D_3_ (*n* = 149)	Placebo (*n* = 100)
Age, years	67.8 (12.0)	64.5 (11.7)	67.9 (10.2)	64.2 (9.2)
Male sex, *n* (%)	37 (57.8)	28 (60.9)	110 (73.8)	62 (62.0)
BMI, kg/m^2^	21.6 (3.3)	21.7 (3.1)	22.2 (3.5)	22.2 (2.7)
Primary tumor site, *n* (%)				
Esophageal	3 (4.7)	4 (8.7)	16 (10.7)	8 (8.0)
Gastric	22 (34.4)	17 (37.0)	68 (45.6)	48 (48.0)
Small intestinal	0 (0.0)	0 (0.0)	1 (0.7)	1 (1.0)
Colorectal	39 (60.9)	25 (54.3)	64 (43.0)	43 (43.0)
Pathological stage, *n* (%)				
Stage I	35 (54.7)	20 (43.5)	64 (43.0)	37 (37.0)
Stage II	15 (23.4)	7 (15.2)	37 (24.8)	36 (36.0)
Stage III	14 (21.9)	19 (41.3)	48 (32.2)	27 (27.0)
Histological type, *n* (%)				
Adenocarcinoma	61 (95.3)	42 (91.3)	133 (89.3)	92 (92.0)
Squamous cell carcinoma	3 (4.7)	4 (8.7)	16 (10.7)	7 (7.0)
Other	0 (0.0)	0 (0.0)	0 (0.0)	1 (1.0)
Baseline 25(OH)D, ng/mL	21.4 (7.9)	21.6 (8.0)	22.9 (8.1)	20.2 (7.7)
FokI genotype, *n* (%)				
CC	64 (100.0)	46 (100.0)	18 (12.1)	6 (6.0)
CT	0 (0.0)	0 (0.0)	99 (66.4)	71 (71.0)
TT	0 (0.0)	0 (0.0)	32 (21.5)	23 (23.0)
Tumor nuclear VDR expression quintile, *n* (%)				
Q1	0 (0.0)	0 (0.0)	49 (32.9)	24 (24.0)
Q2	10 (15.6)	16 (34.8)	26 (17.4)	17 (17.0)
Q3	19 (29.7)	14 (30.4)	24 (16.1)	15 (15.0)
Q4	17 (26.6)	9 (19.6)	30 (20.1)	18 (18.0)
Q5	18 (28.1)	7 (15.2)	20 (13.4)	26 (26.0)

## Data Availability

The datasets generated and/or analyzed during the current study are not publicly available due to ethical restrictions related to patient privacy but are available from the corresponding author on reasonable request and with appropriate institutional approval.

## Supplementary Materials

The following supporting information can be downloaded at https://www.mdpi.com/article/10.3390/cancers18213570/s1: Table S1. Baseline characteristics of randomized participants included in and excluded from the joint biomarker analysis. Table S2. Exploratory analyses by primary tumor site and after excluding colorectal cancer. Table S3. Separate and combined biomarker analyses. Table S4. Event counts, absolute risk differences, and follow-up by treatment and joint biomarker subgroup. Table S5. Inverse probability weighting diagnostics and sensitivity analyses. Table S6. Continuous VDR analyses and model diagnostics.

## Abbreviations

The following abbreviations are used in this manuscript:

BMI	Body Mass Index
CI	Confidence Interval
HR	Hazard Ratio
IU	International Units
25(OH)D	25-Hydroxyvitamin D
