# Supplementary material for: Vitamin D Receptor Genotype, Tumor Expression, and Outcomes After Vitamin D Supplementation in Digestive Tract Cancer: An Exploratory Post Hoc Analysis of the AMATERASU Trial
Source: Cancers (Basel). 2026 Nov 6;18(21):3570. doi: 10.3390/cancers18213570 (PMC13761923; doi:10.3390/cancers18213570)
Supplement: Supplementary file 1 [file cancers-18-03570-s001.zip › cancers-4647864-supplementary.pdf]

Supplementary Materials

Manuscript ID: cancers-4647864

Table S1. Baseline characteristics of randomized participants included in and excluded from the joint biomarker analysis.

Characteristic	Included (n = 359)	Excluded (n = 58)
Age, years, mean (SD)	66.4 (10.6)	65.1 (10.7)
Male sex	237 (66.0)	39 (67.2)
Pathological stage I	156 (43.5)	26 (44.8)
Pathological stage II	95 (26.5)	16 (27.6)
Pathological stage III	108 (30.1)	16 (27.6)
Esophageal cancer	31 (8.6)	9 (15.5)
Gastric cancer	155 (43.2)	19 (32.8)
Small-intestinal cancer	2 (0.6)	0 (0.0)
Colorectal cancer	171 (47.6)	30 (51.7)
Assigned to vitamin D	213 (59.3)	38 (65.5)
Assigned to placebo	146 (40.7)	20 (34.5)

Values are mean (standard deviation) or n (%). Percentages are calculated within each column using 359 included patients and 58 excluded patients as the denominators. Included patients had both FokI genotype and tumor nuclear VDR expression available; excluded patients lacked results for one or both biomarkers. There were no missing values for the characteristics presented among the 417 randomized patients. Comparisons are descriptive and do not establish equivalence or rule out selection bias. SD, standard deviation; VDR, vitamin D receptor.

Table S2. Exploratory analyses by primary tumor site and after excluding colorectal cancer.

A. Patients and events by site, biomarker subgroup, and treatment arm

Site	Biomarker subgroup	Placebo n	Placebo RFS events	Placebo deaths	Vitamin D n	Vitamin D RFS events	Vitamin D deaths
Esophageal	CC/Q2–5	4	1	1	3	0	0
Esophageal	Complement	8	5	2	16	9	8
Gastric	CC/Q2–5	17	4	3	22	2	2
Gastric	Complement	48	8	5	68	12	9
Colorectal	CC/Q2–5	25	10	6	39	5	2
Colorectal	Complement	43	11	5	64	14	10
Small intestinal	Complement	1	0	0	1	0	0

RFS events are relapse or death. The complement comprises all other patients with both biomarkers available. No patient with small-intestinal cancer belonged to the CC/Q2–5 subgroup. Counts do not account for follow-up duration.

B. Unadjusted treatment associations within the CC/VDR Q2–5 subgroup

Site	Outcome	Subgroup n (events)	Treatment HR (95% CI)	P
Colorectal	RFS	64 (15)	0.28 (0.10–0.83)	0.021
Colorectal	OS	64 (8)	0.23 (0.05–1.15)	0.073
Gastric	RFS	39 (6)	0.38 (0.07–2.10)	0.269
Gastric	OS	39 (5)	0.49 (0.08–2.96)	0.441
Esophageal	RFS	7 (1)	Not reported	—
Esophageal	OS	7 (1)	Not reported	—

HRs compare vitamin D with placebo and were obtained from treatment-only Cox models. P values are two-sided Wald P values. The esophageal CC/Q2–5 subgroup had only one RFS event and one death, both in the placebo arm, with no events in the vitamin D arm; stable finite conventional Cox treatment HRs are not reported. All P values and confidence intervals are nominal.

C. Adjusted treatment associations and biomarker subgroup interactions

Population	Outcome	Model n (events)	CC/Q2–5 n (events)	CC/Q2–5 HR (95% CI)	P	Complement HR (95% CI)	Interaction P
Colorectal	RFS	171 (40)	64 (15)	0.42 (0.14–1.24)	0.116	0.89 (0.38–2.11)	0.2761
Colorectal	OS	171 (23)	64 (8)	0.36 (0.07–1.86)	0.224	1.70 (0.55–5.28)	0.1257
Gastric	RFS	155 (26)	39 (6)	0.91 (0.16–5.27)	0.916	0.70 (0.27–1.83)	0.8015
Gastric	OS	155 (19)	39 (5)	1.23 (0.19–8.02)	0.832	0.96 (0.31–3.00)	0.8291
Colorectal excluded	RFS	188 (41)	46 (7)	0.41 (0.07–2.24)	0.302	0.92 (0.44–1.90)	0.4016
Colorectal excluded	OS	188 (30)	46 (6)	0.59 (0.10–3.53)	0.563	1.16 (0.46–2.92)	0.5176

Each model included treatment assignment, joint biomarker subgroup, their interaction, age as a continuous variable, sex, and pathological stage as categorical variables (seven regression coefficients). Each population and endpoint was fitted separately. Treatment HRs in the CC/Q2–5 subgroup were obtained by linear combination of treatment and interaction coefficients; complement HRs are the treatment coefficients with joint = 0. The interaction tests compare treatment effects between biomarker subgroups within the specified population; they do not test treatment-effect differences between tumor sites. The P column reports the treatment test within CC/Q2–5.

The colorectal-exclusion models stratified baseline hazards by the remaining primary tumor sites and included 188 patients: 155 gastric, 31 esophageal, and 2 small-intestinal cancers. The two small-intestinal cancers had no events and contributed no coefficient information through the partial likelihood. The CC/Q2–5 subgroup comprised 46 patients, including 7 RFS events (placebo, 5; vitamin D, 2) and 6 deaths (placebo, 4; vitamin D, 2).

All P values are two-sided Wald P values and all confidence intervals are nominal, without multiplicity correction for these additional analyses. The limited event counts relative to seven coefficients raise concerns about model stability and overfitting. No reliable adjusted esophageal CC/Q2–5 treatment estimate is reported because of the sparse events and zero events in the vitamin D arm. Results are exploratory and do not establish equivalence across sites or benefit outside colorectal cancer.

CI, confidence interval; HR, hazard ratio; OS, overall survival; RFS, relapse-free survival; VDR, vitamin D receptor.

Table S3. Separate and combined biomarker analyses.

A. Patients and events by biomarker combination and treatment arm

Biomarker group	Placebo n	Placebo RFS events	Placebo deaths	Vitamin D n	Vitamin D RFS events	Vitamin D deaths
CT/TT; VDR Q1	18	6	4	31	9	8
CT/TT; VDR Q2–5	76	17	7	100	19	12
CC; VDR Q1	6	1	1	18	7	7
CC; VDR Q2–5	46	15	10	64	7	4

RFS events are relapse or death. The existing VDR quintiles were retained. Counts describe the same 359-patient cohort for both endpoints and all models; they do not account for follow-up duration.

B. Adjusted treatment associations from the full model

Biomarker group	RFS HR (95% CI)	RFS P	OS HR (95% CI)	OS P
CT/TT; VDR Q1	0.65 (0.22–1.96)	0.446	0.83 (0.23–2.94)	0.775
CT/TT; VDR Q2–5	0.86 (0.43–1.68)	0.653	1.41 (0.54–3.64)	0.481
CC; VDR Q1	1.50 (0.18–12.76)	0.712	1.13 (0.13–10.27)	0.911
CC; VDR Q2–5	0.44 (0.18–1.10)	0.079	0.46 (0.14–1.51)	0.203

Treatment HRs compare vitamin D with placebo within each biomarker group. Each full model included treatment, CC versus CT/TT genotype, VDR Q2–5 versus Q1, all three pairwise interactions, and the three-way interaction, plus age, sex, and pathological stage. Baseline hazards were stratified by primary tumor site. There were 11 regression coefficients, 81 RFS events, and 53 deaths. Subgroup HRs were calculated by linear combination of model coefficients.

The one-degree-of-freedom Wald P values for the three-way treatment-by-genotype-by-VDR term were 0.2700 for RFS and 0.3466 for OS. These tests do not constitute an omnibus test that all four subgroup treatment effects are equal. Wide confidence intervals, especially for CC/VDR Q1, limit interpretation. All P values and confidence intervals are nominal; no multiplicity correction was applied to these additional exploratory analyses.

C. Adjusted single-biomarker models

Biomarker	Outcome	Reference-group HR (95% CI)	Other-group HR (95% CI)	Interaction P
FokI genotype	RFS	0.80 (0.45–1.42)	0.63 (0.30–1.32)	0.6179
FokI genotype	OS	1.24 (0.59–2.65)	0.73 (0.30–1.77)	0.3669
VDR category	RFS	0.79 (0.31–2.03)	0.67 (0.39–1.14)	0.7672
VDR category	OS	0.91 (0.31–2.70)	0.87 (0.43–1.76)	0.9424

For the genotype model, the reference group is CT/TT and the other group is CC. For the VDR model, the reference group is Q1 and the other group is Q2–5. Each model included treatment, the single biomarker, their interaction, age, sex, and pathological stage (seven coefficients), with baseline hazards stratified by primary tumor site. HRs compare vitamin D with placebo; interaction P values are one-degree-of-freedom Wald tests.

D. Model fit comparisons using nested partial-likelihood ratio tests

Outcome	Reduced model	LR chi-square	df	P
RFS	Genotype-only	2.96	4	0.5648

Outcome	Reduced model	LR chi-square	df	P
RFS	VDR-only	2.04	4	0.7282
OS	Genotype-only	4.49	4	0.3443
OS	VDR-only	3.16	4	0.5319

Each comparison used the full model in panel B as the larger model and a single-biomarker model in panel C as the nested reduced model, within the same endpoint and cohort. Adjustment covariates, tumor-site stratification, and handling of tied event times were the same across models within each comparison. The full model added four regression coefficients to either reduced model. These are in-sample model fit comparisons; discrimination and externally validated prediction were not evaluated.

All results are exploratory and nominal. The limited events relative to model complexity, and the CC/VDR Q1 placebo cell of six patients with one event for each endpoint, raise concerns about instability and overfitting. Nonsignificant tests do not establish absence of effect modification, equivalence of subgroup effects, or equivalence of models.

CI, confidence interval; df, degrees of freedom; HR, hazard ratio; LR, likelihood ratio; OS, overall survival; RFS, relapse-free survival; VDR, vitamin D receptor.

Table S4. Events and follow-up by joint biomarker subgroup and treatment.

A. Full follow-up event counts and reverse Kaplan–Meier follow-up estimates

Biomarker group	Treatment	n	RFS events	Deaths	Median follow-up years (95% CI)
CC; VDR Q2–5	Placebo	46	15	10	3.92 (3.09–4.95)
CC; VDR Q2–5	Vitamin D	64	7	4	3.92 (3.30–4.93)
Complement	Placebo	100	24	12	3.93 (3.39–4.41)
Complement	Vitamin D	149	35	27	3.93 (3.41–4.84)

RFS events are relapse or death. Counts cover the full observed follow-up and are not restricted to 5 years. The complement includes CC/VDR Q1 and CT/TT at all VDR levels. All 359 patients had both biomarkers available; original randomized treatment assignments were retained.

Median follow-up was estimated from OS times by reverse Kaplan–Meier analysis, with original censoring treated as the event and deaths as censored. Overall median follow-up was 3.93 years (95% CI, 3.77–4.32; n = 359). In the CC/VDR Q2–5 subgroup it was 3.92 years (3.55–4.80; n = 110), and in the complement it was 3.93 years (3.46–4.34; n = 249). These are follow-up estimates, not median survival times.

B. Five-year survival estimates and absolute event risk differences

Biomarker group	Outcome	Placebo survival %	Vitamin D survival %	Event risk difference percentage points (95% CI)	At risk at 5 y P / D
CC; VDR Q2–5	RFS	61.64	86.25	−24.61 (−43.37 to −5.85)	8 / 17
Complement	RFS	71.40	73.45	−2.05 (−15.30 to 11.20)	20 / 33
CC; VDR Q2–5	OS	72.20	91.78	−19.57 (−36.53 to −2.62)	10 / 19
Complement	OS	83.78	77.43	6.35 (−6.31 to 19.02)	26 / 36

Survival probabilities are unadjusted Kaplan–Meier estimates. Event risk is 1 minus survival: relapse or death for RFS and death for OS. Risk differences are vitamin D minus placebo event risk, equivalent to placebo survival minus vitamin D survival; negative values favor vitamin D. Differences and intervals were calculated before rounding survival estimates.

Approximate 95% Wald confidence intervals were calculated as the risk difference plus or minus 1.96 times the square

root of the sum of the independent treatment-arm Greenwood variances. Intervals are pointwise and nominal, with no adjustment for covariates or multiplicity. These are additional exploratory estimates and do not replace the adjusted Cox analyses.

At risk counts are listed as placebo / vitamin D. Only 8–36 patients per group remained at risk at 5 years. Sparse events, limited follow-up, and the large-sample approximation limit precision. The positive complementary-group death risk difference and its wide interval do not exclude clinically important harm. These survival analyses do not establish safety or justify changes in clinical practice.

CI, confidence interval; D, vitamin D; OS, overall survival; P, placebo; RFS, relapse-free survival; VDR, vitamin D receptor; y, years.

Table S5. Inverse probability weighting diagnostics and sensitivity analyses.

A. Clinical characteristics before and after weighting

Characteristic	All randomized<br>n = 417	Complete cases<br>unweighted<br>n = 359	Complete cases<br>IPW<br>n = 359
Age, mean years	66.23	66.42	66.25
Male, %	66.19	66.02	66.18
Female, %	33.81	33.98	33.82
Stage I, %	43.65	43.45	43.62
Stage II, %	26.62	26.46	26.62
Stage III, %	29.74	30.08	29.75
Esophageal cancer, %	9.59	8.64	9.58
Gastric cancer, %	41.73	43.18	41.71
Small-intestinal cancer, %	0.48	0.56	0.48
Colorectal cancer, %	48.20	47.63	48.24
Placebo, %	39.81	40.67	39.64
Vitamin D, %	60.19	59.33	60.36

The 58 excluded patients are compared with the 359 complete cases in Table S1. Here, all randomized patients form the descriptive reference for the weighted complete cases. Percentages are calculated within each column; weighted proportions do not represent additional observed patients. No formal balance significance tests were performed.

Inclusion probabilities were estimated using logistic regression with continuous age and categorical sex, stage, primary tumor site, and treatment assignment. All predictors were observed for all 417 patients. The model used 415 patients because both small-intestinal cases had complete data; these two cases were assigned probability 1 and weight 1 as an explicit convention. Probabilities ranged from 0.6663 to 1.0000. The convention does not establish the population inclusion probability for small-intestinal cancer.

Among 359 complete cases, unstabilized inverse-probability weights had mean 1.1616, median 1.1458, minimum 1.0000, maximum 1.5009, and sum 417.0292. No truncation was applied. Weights address availability of both biomarkers, not treatment assignment or censoring.

B. Adjusted Cox estimates with and without IPW

Outcome	Analysis	CC/VDR Q2–5<br>HR (95% CI)	CC/VDR<br>Q2–5<br>P	Complement<br>HR (95% CI)	Interaction<br>P
RFS	Unweighted	0.44 (0.18–1.10)	0.078	0.87 (0.50–1.50)	0.2113

Outcome	Analysis	CC/VDR Q2–5 HR (95% CI)	CC/VDR Q2–5 P	Complement HR (95% CI)	Interaction P
RFS	IPW	0.43 (0.16–1.12)	0.085	0.88 (0.51–1.50)	0.2020
OS	Unweighted	0.46 (0.14–1.51)	0.199	1.33 (0.65–2.70)	0.1346
OS	IPW	0.44 (0.12–1.59)	0.211	1.34 (0.66–2.69)	0.1349

HRs compare vitamin D with placebo within each biomarker subgroup. All models included treatment, subgroup, their interaction, continuous age, categorical sex and pathological stage, with baseline hazards stratified by primary tumor site. All used 359 actual patients, 81 RFS events, and 53 deaths. The complement comprises CC/VDR Q1 and CT/TT at all VDR levels. The unweighted rows reproduce the primary adjusted analyses.

IPW models used robust sandwich standard errors, treating estimated weights as fixed; uncertainty from fitting the inclusion-probability model was not propagated. Unweighted rows use the original model-based standard errors. Thus, interval differences reflect both weighting and the variance calculation. All subgroup treatment and one-degree-of-freedom Wald interaction P values, and all confidence intervals, are nominal.

Weighted Cox output may display rounded weighted patient and event totals. These are not additional observed patients or events. Small-intestinal cases had no RFS or OS events, so their stratum contributed no coefficient information through the partial likelihood.

IPW relies on an adequate selection model and observed clinical factors sufficiently accounting for selection relevant to the outcomes and biomarker classification. Similar weighted and unweighted estimates do not rule out selection related to unobserved biomarker values or other unmeasured factors, nor establish that selection bias was eliminated. The small-intestinal probability convention and variance-estimation limitations add uncertainty. These post hoc analyses are exploratory; biomarker values were not imputed.

CI, confidence interval; HR, hazard ratio; IPW, inverse probability weighting; OS, overall survival; RFS, relapse-free survival; VDR, vitamin D receptor.

Table S6. Continuous VDR analyses and model diagnostics.

A. Linear VDR models and proportional hazards tests

Outcome	Model	Three-way ratio (95% CI)	Three-way P	Global PH P
RFS	Site stratified; stage adjusted	0.538 (0.196–1.477)	0.2290	0.5164
RFS	Site and stage stratified	0.554 (0.204–1.499)	0.244	0.8858
OS	Site stratified; stage adjusted	0.409 (0.101–1.665)	0.2122	0.5068

All models used 359 complete cases (81 RFS events; 53 deaths), with randomized treatment, CC versus CT/TT genotype, VDR score per 100 points, all pairwise interactions, the three-way interaction, age, and sex. Stage was adjusted categorically or used jointly with tumor site for stratification, as indicated. The three-way ratio is the ratio between genotypes of the per-100-point change in the vitamin D-versus-placebo treatment HR. It is not a treatment HR. Three-way P values are one-degree-of-freedom Wald tests.

Within-genotype changes in treatment HR per 100-point higher VDR

Outcome	CC ratio (95% CI)	CC P	CT/TT ratio (95% CI)	CT/TT P	Joint P
RFS	0.731 (0.329–1.625)	0.442	1.321 (0.737–2.368)	0.350	0.4859
OS	0.627 (0.200–1.969)	0.424	1.532 (0.684–3.430)	0.299	0.4261

RFS ratios use the site-and-stage-stratified model; OS ratios use site stratification and stage adjustment. A ratio below 1 indicates a lower treatment HR at higher VDR, not necessarily a treatment HR below 1. Joint P values test both the treatment-by-continuous-VDR term and the three-way term (2 df), equivalently testing no VDR dependence of treatment HR in either genotype under the linear model.

Schoenfeld residual tests used analysis time. In the site-stratified continuous RFS model, stage III had P = 0.0233. In the site-and-stage-stratified RFS model, all estimable individual terms had P > 0.05. In the continuous OS model, the three-way term had P = 0.0461; other individual terms had P > 0.05. Base and omitted factor levels are not estimable tests. Nonsignificant global or individual tests do not establish proportional hazards. All tests and confidence intervals are nominal.

B. Targeted influence and time dependence assessments

Outcome	Analysis	Patients	Events	Three-way ratio (95% CI)	P
RFS	All cases	359	81	0.554 (0.204–1.499)	0.244
RFS	Omit largest influence	358	80	0.461 (0.164–1.295)	0.142
OS	All cases	359	53	0.409 (0.101–1.665)	0.2122
OS	Omit largest influence	358	52	0.280 (0.058–1.357)	0.114

Influence was assessed on the three-way coefficient in the full linear VDR models: joint site-and-stage stratification for RFS; site stratification and categorical stage adjustment for OS. Approximate case-deletion DFBETAs were divided by the full-model standard error of that coefficient. The maximum absolute standardized values were 0.311977 for RFS and 0.374153 for OS; each maximum identified one case. The respective case was omitted only for the endpoint-specific sensitivity analysis, and all 359 patients were retained in the primary analyses. The two endpoint-specific cases are not assumed to be the same patient.

These ratios are exponentiated three-way coefficients, not treatment HRs. One-case refitting changed their magnitudes; nonsignificance after omission does not show that the cases are uninfluential. These targeted diagnostics do not assess all regression coefficients, the original binary subgroup model, or every possible pattern of case influence.

OS model allowing the three-way coefficient to vary with log time

Assessment	Estimate or statistic	P
Log-time term	0.845 (0.410–1.745)	0.649
Nested model comparison	LR chi-square 0.18; 1 df	0.6749

The time term was treatment × CC × (VDR/100) × ln(time/3 years), added to the full site-stratified OS model with age, sex, and stage adjustment. The ratio in the log-time row is the exponentiated coefficient of this additional term. Centering at 3 years changes its reference time, not the follow-up limit. The time-varying model used 359 patients and 53 deaths; log partial likelihood was −194.24059, compared with −194.32856 for the constant-coefficient model. The Wald P value and nested partial-likelihood ratio P value are distinct tests of the added coefficient.

This targeted log-time alternative did not clearly improve fit, but does not negate the nominal Schoenfeld finding for the OS three-way term (P = 0.0461) or exclude other forms of time dependence. Multiple term-level PH tests were performed, and all these diagnostics are exploratory.

C. Restricted cubic spline sensitivity analyses

Outcome	Model	No. of coeff.	LR chi-square 4 df	Fit P	Wald chi-square 2 df	Three-way P
RFS	Site and stage stratified	13	2.44	0.6547	0.85	0.6531
OS	Site stratified; stage adjusted	15	0.94	0.9182	0.93	0.6288

Restricted cubic splines were constructed in all 359 complete cases, using three knots at VDR scores 55, 180, and 322.5 (0.55, 1.80, and 3.225 on the per-100-point scale), from the default Stata percentile rule. These knots were shared by RFS and OS and were not chosen to optimize an outcome association. Each spline had two basis variables: a linear component and one nonlinear component. Both were included with treatment, genotype, all corresponding pairwise interactions, the two three-way terms, and the clinical adjustment shown above.

RFS spline and linear models had log partial likelihoods −254.02983 and −255.25177; OS values were −193.85672 and −194.32856. Each spline specification added four coefficients to the matching linear model. The 4-df likelihood-ratio test assessed all added nonlinear terms jointly across treatment and genotype combinations. It was not a test solely of nonlinear treatment-effect modification. The 2-df Wald test jointly assessed the two treatment-by-genotype-by-spline terms, allowing both linear and nonlinear genotype differences in VDR-associated treatment effects.

All analyses included 359 patients, with 81 RFS events or 53 deaths. With 13 and 15 estimated coefficients, respectively, spline results are susceptible to imprecision and overfitting. Lack of a statistically clear fit improvement does not establish linearity. Lack of a clear interaction does not exclude effect modification or validate the original quintile cutoff. Spline basis coefficients are not interpreted individually as clinical treatment HRs. These added models were not subjected to a separate full influence or proportional-hazards assessment; the diagnostics in Parts A and B pertain to the linear models.

All P values and confidence intervals are nominal, without further multiplicity adjustment. CI, confidence interval; HR, hazard ratio; LR, likelihood ratio; OS, overall survival; PH, proportional hazards; RFS, relapse-free survival; VDR, vitamin D receptor.
